# Macrophage: Biological Functions, Diseases, and Therapeutic Targets

**DOI:** 10.1002/mco2.70883

**Published:** 2026-07-25

**Authors:** Bihang Sun, Linqing Wen, Shiyun Tang, Lu Liu, Nianzhi Chen

**Affiliations:** ^1^ Department of Medical Oncology & Cancer Institute of Integrative Medicine Shuguang Hospital Shanghai University of Traditional Chinese Medicine Shanghai China; ^2^ State Key Laboratory of Ultrasound in Medicine and Engineering College of Biomedical Engineering Chongqing Medical University Chongqing China; ^3^ Hospital of Chengdu University of Traditional Chinese Medicine Chengdu China; ^4^ State Key Laboratory of Discovery and Utilization of Functional Components in Traditional Chinese Medicine, Chinese Medicine Shanghai Key Laboratory of Complex Prescription and MOE Key Laboratory For Standardization of Chinese Medicines Institute of Chinese Materia Medica Shanghai University of Traditional Chinese Medicine Shanghai China

**Keywords:** M1/M2 activation continuum, M1/M2 imbalance in disease, macrophage polarization, macrophage‐targeted therapeutics, tissue‐resident macrophages

## Abstract

Macrophages are sentinel innate immune cells that arise from embryonic precursors and bone marrow monocytes, displaying a functional continuum that transcends the classical M1 (pro‐inflammatory)/M2 (anti‐inflammatory) dichotomy. Under homeostatic conditions, balanced M1/M2 polarization preserves tissue integrity by coordinating immune surveillance, efferocytosis, and tissue repair. When this equilibrium is disrupted, however, M1‐skewed responses drive chronic inflammation and autoimmunity, whereas M2‐skewed polarization facilitates tumor immune evasion and organ fibrosis. Although diverse therapeutic strategies—including reprogramming, depletion, blockade of monocyte recruitment, CAR‐M cells, and nanomedicine—are being explored to restore homeostasis, clinical translation remains constrained by the lack of pathogenic subset‐specific markers, insufficient predictive biomarkers for patient stratification, species divergence between mice and humans, and the temporal complexity of context‐dependent intervention windows. In this Review, we systematically delineate macrophage plasticity and the molecular mechanisms underlying polarization imbalance, evaluate existing and emerging macrophage‐directed interventions, and dissect these translational bottlenecks in depth, highlighting how single‐cell multi‐omics, humanized models, and dynamic biomarkers can overcome them. By providing a roadmap for precisely calibrating macrophage functional states and restoring M1/M2 balance, this framework will accelerate the development of precision immunotherapies aimed at re‐establishing immune homeostasis across a broad spectrum of human pathologies.

## Graphical abstract

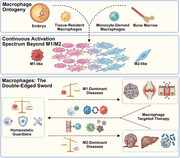

## 1 Introduction

Since their initial description as professional phagocytes by Elie Metchnikoff in the late 19th century, our understanding of macrophage biology has undergone a fundamental transformation [1, 2]. Macrophages were traditionally regarded as terminally differentiated constituents of the mononuclear phagocyte system, functioning primarily as scavengers that eliminate pathogens and clear cellular debris [3]. The past two decades, however, have witnessed two pivotal paradigm shifts that have redefined this view. First, developmental fate‐mapping studies have overturned the long‐standing dogma that all macrophages arise from bone marrow monocytes, demonstrating instead that most tissue‐resident populations are seeded by embryonic precursors from the yolk sac or fetal liver and maintained through local self‐renewal [4, 5]. Second, the growing appreciation of macrophage functional plasticity has dissolved the rigid M1 (pro‐inflammatory)/M2 (anti‐inflammatory) dichotomy established by in vitro polarization experiments [6]. Single‐cell multi‐omics technologies now resolve, at unprecedented resolution, a continuous spectrum of activation states dynamically sculpted by tissue microenvironmental cues [7, 8]. This has revealed a breadth of functional heterogeneity and phenotypic diversity that far exceeds the explanatory power of traditional classification frameworks.

The fragmentation of current knowledge and the urgent need for macrophage‐targeted precision immunotherapy together motivate this systematic examination of macrophage biology, disease associations, and therapeutic strategies. Macrophages are broadly implicated in the pathogenesis of major human diseases—including cancer, autoimmune disorders, metabolic diseases, fibrosis, and infectious diseases—and their central role as disease drivers has been extensively validated [9, 10]. However, although macrophage‐directed interventions have entered clinical development, their clinical translation is hampered by the off‐target toxicity of depletion approaches, the variable efficacy of reprogramming strategies, and a translational gap stemming from species divergence [11, 12]. A comprehensive synthesis is thus required to elucidate how macrophage ontogeny, polarization, and function interact to drive distinct disease mechanisms, thereby establishing a theoretical framework for the development of targeted therapies. We first systematically examine the developmental origins, polarization heterogeneity, and metabolic regulation of macrophages. We then focus on their three core homeostatic functions—endogenous clearance, immune surveillance, and tissue repair—and delineate how these functions govern the transition from tissue homeostasis to pathological progression. Next, we provide an in‐depth analysis of the mechanisms underlying macrophage dysfunction in cancer, autoimmune diseases, metabolic disorders, fibrosis, and infectious diseases. We then critically evaluate current macrophage‐targeted therapeutic strategies, including depletion, reprogramming, recruitment blockade, and cell‐ and nanomaterial‐based therapies, and dissect their translational bottlenecks. Building on this foundation, we highlight future directions such as single‐cell atlas‐guided identification of pathogenic subsets, the establishment of dynamic surveillance biomarkers, and the development of humanized models, with the aim of providing a theoretical framework and a strategic roadmap for precision immunotherapy that restores immune homeostasis.

A central tenet of this review is the dialectical framing of macrophages as a “double‐edged sword.” Rather than resorting to the rigid labels of “beneficial” and “harmful,” we attribute their functional consequences to the degree of congruence between a given activation state and the specific pathological context. When the precise coordination between microenvironmental signals and macrophage responses breaks down, homeostatic guardians are transformed into disease drivers [13]. This conceptual reframing not only deepens our understanding of macrophage biology but also charts a new course for therapeutic modulation of macrophage function to treat major human diseases.

## 2 Origin, Heterogeneity, and Activation States

Macrophages are innate immune cells distributed throughout virtually all tissues and body cavities [14, 15], representing a highly heterogeneous population shaped jointly by developmental origin, the tissue microenvironment, and metabolic state [16]. Although long considered to arise exclusively from bone marrow monocytes, fate‐mapping studies have revealed that most tissue‐resident macrophages instead originate from embryonic precursors in the yolk sac or fetal liver and are maintained through local self‐renewal, whereas bone marrow–derived populations are recruited predominantly during inflammatory responses [4, 5]. This developmental duality imparts distinct functional biases and constitutes a fundamental dimension of macrophage heterogeneity. Single‐cell multi‐omics technologies have further demonstrated that macrophages in vivo exist along a continuous activation spectrum whose complexity far exceeds the explanatory framework of the classical M1/M2 dichotomy, and the widespread existence of intermediate and hybrid phenotypes is now well recognized [17, 18, 19]. Concurrently, the emergence of immunometabolism has established that metabolic reprogramming is not a passive consequence of polarization but rather an instructive driver that actively shapes macrophage functional identity [20, 21, 22]. This chapter systematically examines how macrophages integrate their intrinsic lineage identity with extrinsic microenvironmental signals to generate their complex functional spectrum, and is organized around three thematic axes: developmental origins, activation heterogeneity, and immunometabolism.

### 2.1 Developmental Origins

**FIGURE 1:**
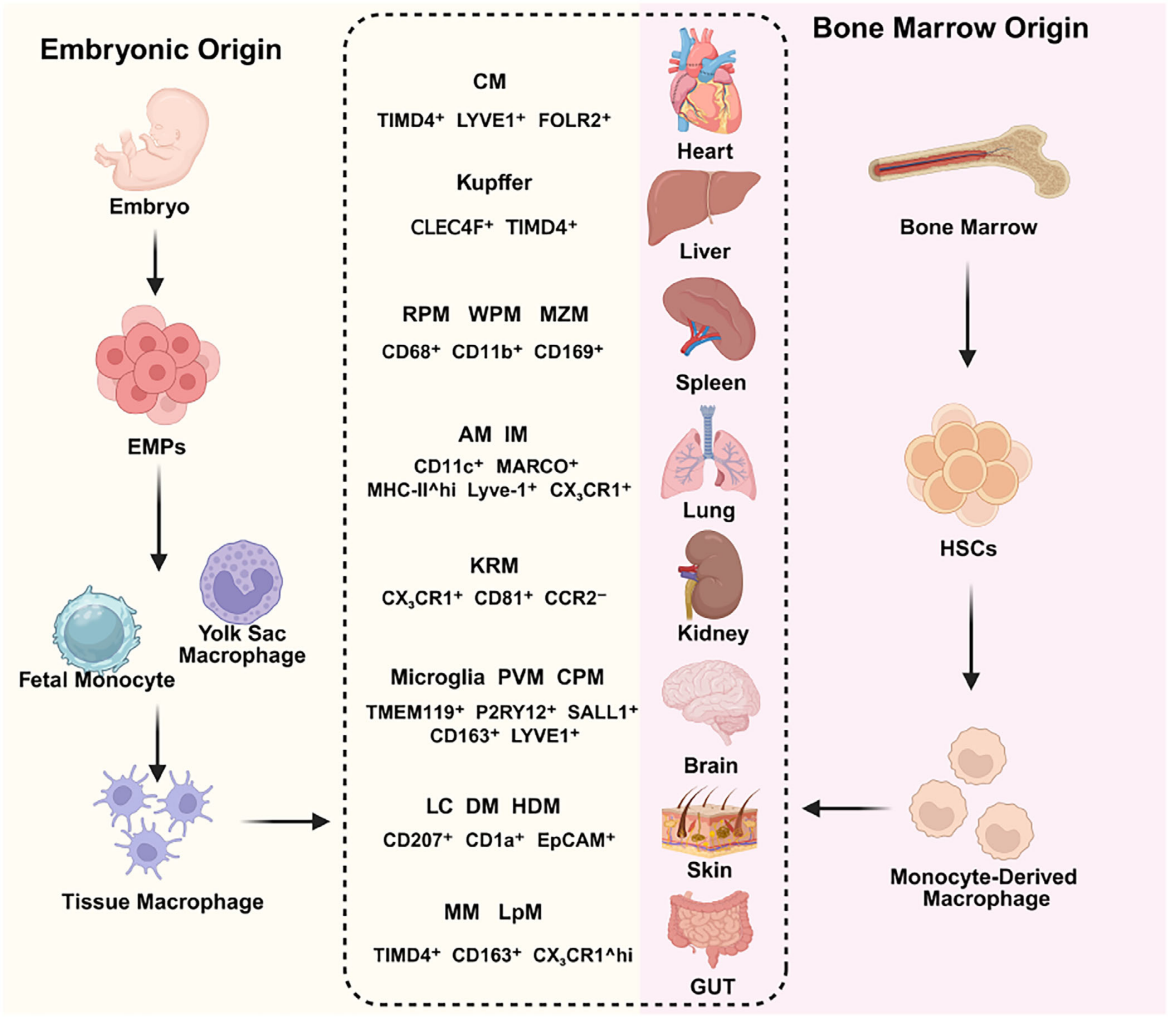
Macrophages derive from either embryonic or bone marrow sources, generating distinct tissue‐resident and recruited populations. Left panel (embryonic origin): Yolk sac–derived macrophages and fetal monocytes (originating from erythromyeloid progenitors) seed developing tissues and differentiate into self‐renewing tissue‐resident macrophages—including microglia, Kupffer cells, alveolar macrophages, interstitial macrophages, splenic macrophages (red pulp, white pulp, marginal zone), kidney resident macrophages, Langerhans cells, dermal macrophages, and gut macrophages (lamina propria and muscularis). Right panel (bone marrow origin): Postnatally, hematopoietic stem cells give rise to circulating monocytes that infiltrate tissues and, under homeostatic or inflammatory conditions, differentiate into monocyte‐derived macrophages. EMPs, erythro‐myeloid progenitors; HSCs, hematopoietic stem cells; CM, cardiac macrophages; RPM, red pulp macrophage; WPM, white pulp macrophage; MZM, marginal zone macrophage; AM, alveolar macrophage; IM, interstitial macrophage; KRM, kidney resident macrophage; PVM, perivascular macrophage; CPM, choroid plexus macrophage; LC, Langerhans cell; DM, dermal macrophage; HDM, hypodermal macrophage; MM, muscularis macrophage; LpM, lamina propria macrophage. (Created in https://BioRender.com).

Macrophages were first described by Karl Wilhelm von Kupffer in 1876 and subsequently identified and characterized by Tadeusz Browicz approximately two decades later. The traditional view held that all tissue macrophages arise from bone marrow hematopoietic stem cells, a concept that formed the cornerstone of the mononuclear phagocyte system. Owing to technical limitations, however, the precise characterization of distinct macrophage populations and their specific markers long remained elusive [23, 24]. In recent years, the widespread application of high‐resolution fate‐mapping and single‐cell multi‐omics technologies has revealed that macrophages in vivo have a dual origin, comprising embryonic‐derived populations and bone marrow‐derived populations (Figure **1**) [25, 26]. During the earliest stages of embryonic development, primitive hematopoiesis in the yolk sac generates the first macrophages, which subsequently migrate to and colonize developing tissues, thereby establishing the tissue‐resident macrophage pool [27, 28]. For instance, microglia in the brain originate almost exclusively from the yolk sac and are maintained throughout life within the central nervous system through local self‐renewal [29, 30]. As embryonic development proceeds, the center of hematopoiesis shifts from the yolk sac to the fetal liver, where hematopoietic stem cells give rise to an additional wave of macrophage precursors that similarly migrate to and seed developing organs, collectively forming the embryonic‐derived resident macrophage compartment [31]. After birth, hematopoiesis transitions to the bone marrow, where hematopoietic stem cells differentiate into monocytes that are released into the circulation [32]. Under both homeostatic and inflammatory conditions, these monocytes enter tissues and differentiate into macrophages, commonly referred to as recruited macrophages [33].

Developmental origin fundamentally shapes the biological properties and functional roles of macrophages. Embryonically derived tissue‐resident macrophages differ markedly from their bone marrow‐derived counterparts in both developmental lineage and functional attributes [34, 35, 36]. Resident macrophages establish stable contacts with specialized tissue cells and, by sensing and integrating local and systemic signals, provide growth factors to parenchymal cells, participate in nutrient recycling, and clear metabolic waste, thereby playing essential roles in tissue development, homeostasis, and repair [37]. These cells are highly specialized, with their transcriptional profiles and functions being profoundly shaped by the tissue microenvironment, rendering them indispensable components of organ structure and function [38]. Under certain pathological conditions, however, resident macrophages may also contribute to metabolic dysregulation, inflammation, and tumorigenesis [39, 40]. In contrast, bone marrow‐derived macrophages enter tissues only at low levels under physiological conditions to replenish the resident pool, but are recruited in large numbers to sites of inflammation or injury, where they rapidly differentiate [41, 42]. Their phenotypes and functions are driven more directly by local microenvironmental cues, endowing them with greater plasticity and context dependence.

In summary, developmental origin fundamentally shapes the core functional properties of macrophages and constitutes a critical foundation for their profound heterogeneity [43, 44]. Embryonically derived microglia maintain neuronal connections and participate in synaptic pruning [45], whereas Kupffer cells efficiently clear blood‐borne substances while promoting immune tolerance—specialized functions not readily assumed by bone marrow‐derived macrophages [46]. In diverse pathological settings, macrophages of distinct origins exert divergent, even opposing, regulatory roles: tissue‐resident populations generally preserve protective homeostatic functions, whereas bone marrow‐derived recruited populations more frequently drive pro‐inflammatory and pro‐tumorigenic responses [47]. For example, in obesity, protective resident macrophages are functionally suppressed while pathogenic bone marrow‐derived populations undergo massive expansion [48]; during tumor progression, bone marrow‐derived tumor‐associated macrophages (TAMs) with pro‐tumorigenic properties dominate the macrophage compartment [49]. Thus, developmental origin serves not only as a basis for lineage tracing in developmental biology but also as a fundamental dimension for understanding macrophage functional polarization and phenotypic plasticity in health and disease.

### 2.2 The Classical M1/M2 Dichotomy and Beyond

Macrophage polarization describes the spectrum of functional activation states that macrophages adopt in response to specific microenvironmental signals [50, 51], a highly plastic process that participates extensively in both physiological and pathological contexts. Elucidating the molecular mechanisms governing polarization and causally linking distinct polarization programs to their downstream signaling pathways and associated physiological or pathological outcomes, therefore, constitutes a central objective in macrophage biology.

**FIGURE 2:**
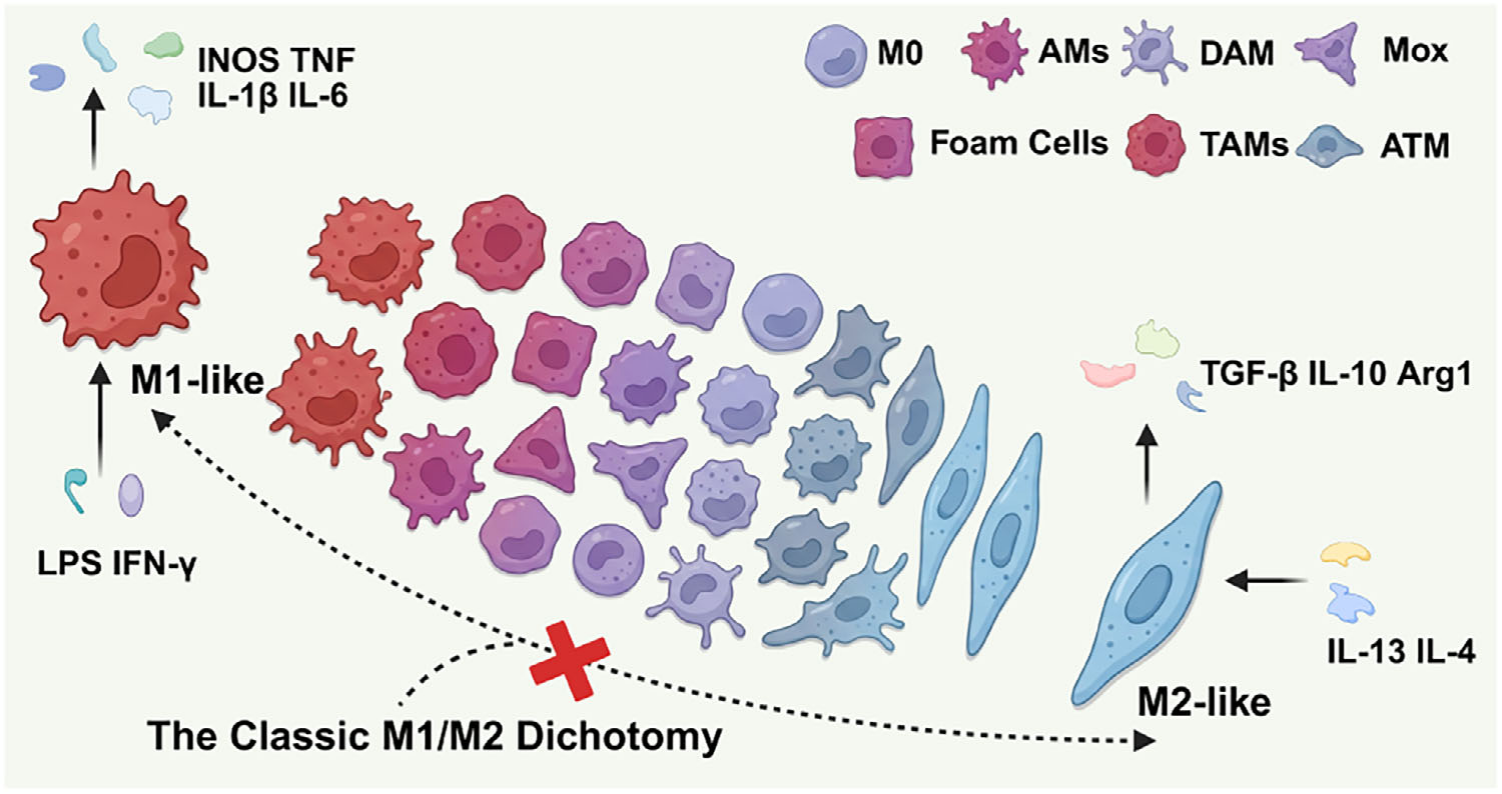
A Spectrum model of macrophage activation transcends the classical M1/M2 dichotomy. Historically, resting macrophages (M0) stimulated with LPS and IFN‐γ adopt an M1‐like phenotype expressing iNOS, TNF, IL‐1β, and IL‐6, whereas IL‐4 or IL‐13 drives an M2‐like phenotype marked by TGF‐β, IL‐10, and Arg1. However, although this linear M1–M2 axis has heuristic value, it is now widely considered an oversimplification. In vivo, macrophage activation instead constitutes a multidimensional continuum, where hybrid phenotypes and the co‐expression of canonical M1 and M2 markers produce functional states that defy binary classification. AMs: alveolar macrophages; DAM: disease‐associated microglia; Mox: Mox macrophages; TAMs: tumor‐associated macrophages; ATM: adipose tissue macrophages. (Created in https://BioRender.com).

The classical M1/M2 dichotomy, largely derived from in vitro polarization experiments using defined stimuli, represents a substantial oversimplification of macrophage phenotypes observed in vivo [52, 53]. Stimulation with interferon‐γ (IFN‐γ) or bacterial lipopolysaccharide (LPS) drives macrophages toward an M1‐polarized state, endowing them with pro‐inflammatory, microbicidal, and antitumor functions [54, 55]. These cells produce high levels of inflammatory cytokines such as tumor necrosis factor (TNF), interleukin‐1 (IL‐1), and interleukin‐6 (IL‐6), efficiently recruit other immune cells, and possess potent pathogen‐killing capacity [56, 57]; their excessive activation, however, can inflict collateral tissue damage. Conversely, exposure to interleukin‐4 (IL‐4) or interleukin‐13 (IL‐13) induces M2 polarization, conferring anti‐inflammatory, pro‐repair, and pro‐fibrotic properties. M2‐polarized macrophages secrete anti‐inflammatory mediators such as interleukin‐10 (IL‐10), promote angiogenesis and tissue remodeling, and facilitate wound healing by clearing apoptotic cells [58, 59], yet their persistent skewing can also drive pathological fibrosis or foster tumor progression. Although this binary framework has served as a useful conceptual scaffold, macrophages in the in vivo environment exhibit a breadth of heterogeneity and plasticity that extends far beyond its explanatory power. Consequently, the canonical M1 and M2 states are more accurately viewed as two extremes of a continuous activation spectrum [60, 61], across which functional states transition dynamically rather than residing in fixed, discrete categories (Figure **2**). For instance, within the tumor microenvironment, tumor‐associated macrophages display a functionally mixed, immunosuppressive phenotype that is neither classically M1 nor fully M2 [62, 63]. In atherosclerosis, the metabolic reprogramming of foam cells defines a distinct pathological state that does not conform to the M1/M2 classification [64, 65]. Likewise, adipose tissue macrophages in obesity‐related metabolic syndrome form a continuum co‐expressing both M1 and M2 markers (Table **1**) [66, 67]. These observations illustrate that the M1/M2 dichotomy not only fails to accommodate hybrid phenotypes but also overlooks the inherent heterogeneity of tissue‐resident macrophages and the functional biases conferred by their developmental origins.

**TABLE 1:** Macrophage phenotypes and markers.

Cell type	Disease/Tissue context	Phenotypic features	Key markers	References
DAM	Alzheimer's disease and other neurodegenerative disorders	Exhibiting a mixed phenotype	TREM2^+^, CD11c^+^, CD206^+^, ApoE^+^, Clec7a^+^, Lpl^+^	[68, 69]
AMs	COPD/Pulmonary fibrosis	M1/M2 dual‐positive mixed phenotype	CD163^+^, CD86^+^, CD206^+^, FABP4^+^	[70]
Mox	plaque Atherosclerotic s	Independent of the M1/M2 dichotomy	HO‐1^+^, Txnrd1^+^, Nrf2^+^, IL‐1β^+^	[70, 71]
TAMs	Multiple solid tumors	Encompassing M1‐like, M2‐like, and independent subsets	CD68^+^, CD163^+^, TREM2^+^, SPP1^+^, FOLR2^+^, MARCO^+^, PD‐L1^+^	[68, 71]
ATMs	Obesity and metabolic syndrome	Comprising mixed phenotypes and metabolically specialized subsets	M1‐like: CD11c^+^, iNOS^+^, TNF‐α^+^; M2‐like: CD206^+^, IL‐10^+^; LAMs: TREM2^+^, CD9^+^; MMe: ABCA1^+^, CD36^+^, PLIN2^+^	[70]

*Note*: The phenotypes and markers of the subsets described above are not mutually exclusive; a single cell may simultaneously express multiple markers, reflecting the high degree of macrophage plasticity in vivo.

Abbreviations: ApoE, apolipoprotein E; COPD, chronic obstructive pulmonary disease; HO‐1, heme oxygenase‐1; Txnrd1, thioredoxin reductase 1; LAMs, lipid‐associated macrophages; MMe, metabolically activated macrophages.

In summary, while the classical M1/M2 framework has provided an essential starting point for polarization research, it is fundamentally insufficient to capture the full complexity of macrophage biology in vivo [72]. Single‐cell multi‐omics technologies are now resolving, at unprecedented resolution, a continuous spectrum of macrophage activation states, consistently revealing the widespread existence of intermediate and hybrid phenotypes [73]. These advances establish a new conceptual foundation for moving beyond the classical paradigm and informing the rational design of precision‐targeted therapeutic strategies.

### 2.3 Decoding Heterogeneity: Insights From Single‐Cell Technologies

The widespread application of single‐cell multi‐omics technologies has fundamentally reshaped our understanding of macrophage heterogeneity [69, 74]. Rather than constituting a uniform entity, macrophages represent a highly specialized cell population whose identity is profoundly shaped by the resident tissue microenvironment. Macrophages in different organs adapt to local cues and acquire highly specialized functions: microglia in the brain participate in synaptic pruning and continuously survey neural activity, Kupffer cells in the liver clear senescent erythrocytes and foreign particles from blood, osteoclasts in bone are dedicated to bone resorption, and alveolar macrophages maintain the sterility of the gas exchange surface by phagocytosing inhaled particles and pathogens [75, 76]. However, macrophage heterogeneity extends far beyond interorgan variation. Even within a single tissue, multiple subpopulations with distinct functional properties and anatomical localizations coexist [77, 78, 79]. In the liver, for instance, liver capsular macrophages and central vein‐associated macrophages coexist alongside Kupffer cells; in the lung, interstitial macrophages complement alveolar macrophages; and up to seven distinct resident macrophage subsets have been characterized in the mouse kidney. Recent work has further demonstrated that Kupffer cells can be stratified by differential expression of CD206 and endothelial cell‐selective adhesion molecule (ESAM) into two populations: a dominant CD206^−^ESAM^−^ Kupffer cell subset 1 (KC1) subset and a minor CD206^+^ESAM^+^ Kupffer cell subset 2 (KC2) subset [80, 81]. In the lung, major histocompatibility complex class II (MHCII)^+^CD206^−^ interstitial macrophages are predominantly localized to interstitial regions adjacent to alveoli, whereas their CD206^+^MHCII^−^ counterparts are more broadly distributed throughout the peribronchial interstitium [82]. Traditional bulk tissue homogenate sequencing captures only population‐averaged signals, inevitably masking cell‐to‐cell heterogeneity. Single‐cell RNA sequencing now resolves subset composition and specific markers at single‐cell resolution, enabling robust discrimination between tissue‐resident and recruited macrophages and further facilitating fine‐grained subset classification [83, 84]. For instance, pan‐cancer analyses have identified two subsets of tumor‐infiltrating monocytes (CD14^+^ and CD16^+^), a lymphatic vessel endothelial hyaluronan receptor 1 (LYVE1)^+^ interstitial macrophage subset, and seven TAM subsets [85, 86]. Another study employing single‐cell regulatory network inference and clustering (SCENIC) identified classical monocytes, nonclassical monocytes, and five TAM subsets across multiple cancer types [87]. As research advances, numerous previously unrecognized macrophage subsets of remarkable heterogeneity continue to be uncovered. Single‐cell technologies have substantially deepened our understanding of the multilayered regulation of macrophage diversity—a diversity shaped not only by dynamic extrinsic microenvironmental signals, but also profoundly influenced by the intrinsic lineage identity conferred by embryonic or monocytic origin. Ongoing and future studies, leveraging cutting‐edge single‐cell multi‐omics approaches, aim to map the continuous functional landscape of macrophages at increasingly higher resolution and to redefine subset identity through an integrated framework that incorporates tissue context, activation signals, and transcriptomic signatures.

### 2.4 Immunometabolism: Fueling Functional Plasticity

The defining feature of macrophages is their remarkable functional plasticity [88]. In response to constantly changing physiological and pathological cues within the tissue microenvironment, macrophages switch their polarization states, enabling dynamic transitions in both phenotype and function [89]. During this adaptation, metabolic reprogramming serves as a key mechanism driving functional plasticity by reshaping core metabolic pathways and generating metabolites that support specific functional programs [90, 91]. The rapid advance of immunometabolism over the past decade has revealed that alterations in intracellular metabolic fluxes during immune cell activation can actively program cellular functional states. In other words, macrophage functional orientation is not solely dictated by upstream signals but is co‐determined by concomitant metabolic reprogramming [92]. Metabolic state thus constitutes both a prerequisite for and a consequence of functional execution. The metabolic network and immune function of macrophages form a bidirectional regulatory circuit: on the one hand, macrophages directly modulate their own functional state and polarization trajectory through dynamic remodeling of core metabolic pathways [93, 94]; on the other, microenvironment‐driven polarization shifts reciprocally reshape the metabolic network to meet corresponding energetic and biosynthetic demands [95]. This deep metabolic–functional coupling precisely governs core macrophage activities—including inflammatory cytokine release, antigen presentation, and tissue repair—by altering the abundance of key metabolites, modulating signaling pathway activity, and influencing epigenetic modifications.

Cellular metabolism, a complex network of catabolic and anabolic reactions, is fundamental to homeostasis [96]. Metabolic substrates are processed through diverse biochemical pathways into intermediate metabolites, which either fuel mitochondrial oxidation to provide energy and biosynthetic precursors or are promptly exported from the cell to avert toxic accumulation. This metabolic regulatory framework is highly specialized in immune cells and is particularly prominent in macrophages [97, 98]. Immunometabolism research has unequivocally demonstrated that cellular metabolism plays a central role in sustaining macrophage function and plasticity. Although metabolic changes were traditionally viewed as primarily serving adenosine triphosphate (ATP) homeostasis, macrophage metabolic reprogramming extends far beyond this role, participating critically in lipid synthesis, nucleotide biosynthesis, cell signaling, and gene expression.

The metabolic profile of M1 macrophages is characterized by enhanced glycolysis, activated pentose phosphate pathway (PPP) activity, increased fatty acid synthesis, and a truncated tricarboxylic acid (TCA) cycle with impaired oxidative phosphorylation [68, 70, 99]. Upon Toll‐like receptor stimulation, glucose uptake and glycolytic flux increase, and the accumulation of glycolytic intermediates fuels the PPP, providing reduced nicotinamide adenine dinucleotide phosphate (NADPH) for nucleotide synthesis and reactive oxygen species production [100]. Enhanced glycolysis is accompanied by elevated lactate production [101]; pyruvate entry into the TCA cycle is followed by cycle breaks at isocitrate dehydrogenase and succinate dehydrogenase (SDH), where excessive succinate accumulation stabilizes hypoxia‐inducible factor 1α, further suppressing mitochondrial respiration and reinforcing the glycolytic phenotype [102]. M1 macrophages highly express inducible nitric oxide synthase, metabolizing arginine to produce nitric oxide. In contrast, M2 macrophages rely predominantly on fatty acid oxidation and oxidative phosphorylation, exhibiting enhanced mitochondrial respiration with an intact TCA cycle that efficiently supplies substrates to the electron transport chain [103, 104]. M2 macrophages highly express arginase‐1, and arginine metabolism through this pathway determines their anti‐inflammatory orientation [105, 106]. Energy metabolism thus exhibits a striking division of labor: glycolysis, though relatively inefficient in ATP yield, responds rapidly to meet the immediate demands of M1 cells, whereas oxidative phosphorylation, despite its slower rate, achieves higher efficiency, supporting the sustained reparative and anti‐inflammatory functions of M2 cells [107].

Thus, the functional output of macrophages in vivo reflects the integration of intrinsic heterogeneity imposed by developmental origin and functional plasticity driven by the tissue microenvironment. This complex interplay not only establishes macrophages as central regulators of organismal homeostasis but also determines their dual roles across diverse disease processes. The direct coupling between immunometabolism and functional state provides a robust theoretical foundation for therapeutically modulating immune responses by targeting metabolic pathways [108, 109]. Accordingly, in pathological contexts such as atherosclerosis, obesity‐related metabolic diseases, autoimmunity, and cancer, targeting macrophage immunometabolism to modulate their functional plasticity has emerged as a promising strategy for inhibiting pathogenic functions and reshaping the immune microenvironment.

## 3 Biological Functions: From Homeostasis to Pathogenesis

Macrophages are distributed throughout virtually all tissues and organs and serve as central regulators of organismal homeostasis—acting not only as executors of immune effector functions but also as active sculptors and coordinators of the tissue microenvironment [110, 111]. Their functional repertoire spans multiple domains, including endogenous waste clearance, defense against exogenous threats, and tissue repair [14, 112, 113]. Under physiological conditions, macrophages continuously engulf apoptotic cells, cellular debris, and metabolic waste to preserve tissue homeostasis [114, 115]; this clearance and recycling function represents their most fundamental physiological role. Building upon this foundation, macrophages constitute the first line of innate immune defense [116, 117], constantly surveying the surrounding microenvironment, detecting exogenous threats such as pathogen invasion or malignant transformation, and thereby establishing an immunological surveillance barrier. Simultaneously, they actively promote immune tolerance mechanisms that prevent autoreactive responses and preserve local immune equilibrium [118, 119]. Following tissue injury, macrophages rapidly initiate repair programs, secreting an array of growth factors and enzymes that facilitate wound healing and contribute to the renewal and maintenance of tissue architecture [120]. These core functions are not executed in isolation but are orchestrated by a tightly regulated signaling network, in which efferocytosis‐associated signals, pattern recognition receptor pathways, and key cytokine axes such as transforming growth factor‐β (TGF‐β) collectively determine macrophage functional orientation and effector output. When microenvironmental signals become dysregulated or these regulatory networks break down, however, macrophages transition from homeostatic guardians to disease drivers [14, 121, 122]. For example, aberrant clearance can lead to atherosclerosis and neurodegeneration, disrupted immune surveillance can precipitate autoimmunity or tumor immune evasion, and uncontrolled repair programs can drive pathological fibrosis. This chapter first systematically delineates the three core homeostatic functions of macrophages and then provides an in‐depth analysis of the key signaling pathways governing these functions, thereby revealing the molecular basis underlying the transition from physiological function to pathological progression.

### 3.1 Homeostasis Functions

#### 3.1.1 Endogenous Clearance and Recycling

Endogenous clearance and recycling constitute a fundamental homeostatic function through which macrophages act as key organizers of the tissue microenvironment [123, 124]. Rather than serving as a simple waste disposal system, this function embodies a homeostatic mechanism that intimately couples catabolic breakdown with resource reclamation. By recognizing, engulfing, and degrading aberrant materials as well as senescent and apoptotic cells, macrophages eliminate disruptive elements from the tissue microenvironment—thereby preserving immune homeostasis—while reintegrating the resulting basic constituents into systemic metabolic networks, enabling efficient nutrient recycling [125, 126].

Efficient clearance of dying cells is a fundamental physiological process required for the maintenance of homeostasis in multicellular organisms. Macrophages, as professional phagocytes, orchestrate the recognition and clearance of apoptotic cells through efferocytosis—a multistep program that simultaneously reclaims cellular constituents [127]. Given that billions of cells undergo apoptosis daily during mammalian embryonic development and adult tissue turnover, failure to promptly clear dying cells results in the release of intracellular contents that activate inflammatory pathways and damage adjacent healthy tissue [128, 129]. The integrity of efferocytosis, therefore, holds fundamental importance for the maintenance of tissue homeostasis. Phagocytosis, the most extensively studied core macrophage function, provides the mechanistic basis for this clearance and recycling activity. By internalizing and degrading endogenous aberrant materials—including misfolded proteins, oxidized lipids, and cellular debris—as well as senescent and apoptotic cells, macrophages eliminate disruptive factors from the tissue microenvironment and reintegrate the liberated basic constituents into metabolic cycles, thereby achieving efficient nutrient recycling and reuse. In the bone marrow, liver, and spleen, macrophages serve as critical regulators of the hematopoietic microenvironment, overseeing the entire erythrocyte production cycle. They clear extruded nuclei during erythroblast enucleation and remove senescent erythrocytes, efficiently recycling iron, amino acids, and bilirubin precursors directly to nascent erythrocytes for hemoglobin synthesis, thus maintaining the dynamic equilibrium of erythropoiesis. In bone, osteoclasts—specialized macrophages—acidify the local microenvironment via vacuolar‐type proton pumps, dissolving hydroxyapatite and degrading the collagen matrix [130]. The liberated calcium, phosphate, and organic components are either utilized for local bone formation or delivered into the systemic circulation, playing an indispensable role in skeletal homeostasis.

However, overload or dysregulation of endogenous clearance and recycling can directly precipitate the collapse of tissue homeostasis and drive the initiation and progression of multiple diseases [131]. In atherosclerotic lesions, macrophages within the vessel wall excessively engulf oxidized low‐density lipoprotein and transform into lipid‐laden foam cells; their eventual death releases pro‐inflammatory contents that further exacerbate plaque inflammation and instability, thereby disrupting vascular homeostasis. In Alzheimer's disease and related neurodegenerative disorders, the capacity of microglia to clear misfolded proteins such as β‐amyloid progressively declines with disease advancement, leading to the accumulation of toxic protein aggregates that trigger neuronal injury and chronic inflammation, driving the neural microenvironment away from homeostasis [132, 133]. Collectively, the clearance and recycling functions executed by macrophages constitute not only a cornerstone of physiological tissue homeostasis but also a critical regulatory node that shapes the pathological trajectory across diverse disease processes.

#### 3.1.2 Immune Surveillance and Tolerance

The efficient clearance of endogenous waste by macrophages epitomizes their core capacity as guardians of tissue homeostasis. However, upon encountering exogenous threats such as pathogen invasion or malignant transformation, macrophages rapidly switch their functional program, transitioning from homeostatic guardians into executors of immune surveillance. As a critical frontline defense of the innate immune system, macrophages continuously survey the host to discriminate self from non‐self and, upon target recognition, mount a rapid response characterized by directed migration and phagocytic elimination of invading pathogens. Activated macrophages subsequently present antigens to the adaptive immune system, thereby triggering more precise, antigen‐specific defense responses [134]. Notably, the immune surveillance function of macrophages is predominantly mediated by the M1‐polarized phenotype [135], which is equipped with potent pro‐inflammatory and microbicidal capacity, whereas M2 macrophages largely lack such effector functions. In the context of intracellular infection, M1 polarization drives the release of inflammatory mediators that execute both pathogen clearance and immune surveillance [136]. The accompanying inflammatory response, however, exerts a dual effect: moderate activation effectively limits pathogen dissemination, while excessive activation inflicts collateral tissue damage and disrupts immune tolerance [137, 138]. As professional antigen‐presenting cells, macrophages not only prime adaptive immunity under inflammatory conditions but also preserve self‐tolerance under homeostatic conditions by presenting self‐antigens to induce T cell anergy or promote regulatory T cell differentiation [139]. Thus, the core function of macrophages lies in dynamically maintaining a delicate balance between immune surveillance and immune tolerance—efficiently recognizing and eliminating foreign threats while strictly avoiding detrimental immune reactions against self‐tissues. The fine equilibrium between these two forces collectively determines the ultimate trajectory of the immune response.

When this balance is disrupted, disease progression diverges along fundamentally opposing paths. In autoimmune diseases such as rheumatoid arthritis, synovial macrophages become aberrantly activated, breaking self‐tolerance and activating autoreactive lymphocytes, culminating in tissue destruction. Similarly, in interferonopathies and macrophage activation syndrome, macrophages adopt a hyperinflammatory state, releasing massive quantities of inflammatory cytokines that drive multiorgan damage affecting the skin, nervous system, and beyond [140, 141]. Conversely, within the tumor microenvironment, excessive immunosuppressive signals endow tumor‐associated macrophages with potent immunosuppressive capacities that suppress the activation and cytotoxic function of CD8^+^ T cells, thereby creating conditions favorable for tumor immune evasion. Thus, the dynamic balance between immune surveillance and immune tolerance serves not only as the central fulcrum through which macrophages maintain tissue homeostasis but also as a critical regulatory interface governing the trajectory of inflammatory diseases and tumor immune escape.

#### 3.1.3 Tissue Remodeling and Repair

Tissue repair and regeneration represent essential biological processes for maintaining organismal homeostasis, encompassing angiogenesis, proliferation of parenchymal cells and fibroblasts, and extracellular matrix deposition and remodeling—all critical for preserving organ structural integrity and physiological function. Although multiple cell types contribute to the repair response, macrophages, by virtue of their remarkable functional plasticity, serve as central regulators at every stage of wound healing and fibrosis, a role validated across diverse model organisms from salamanders and zebrafish to neonatal mice [142, 143].

Upon tissue injury, damage‐associated molecular patterns released from dying cells and pathogen‐associated molecular patterns from invading microorganisms activate macrophages, triggering an inflammatory response that initiates the repair program [144]. During the early phase of injury, macrophages debride the wound by phagocytosing dead cells and tissue debris, while simultaneously serving as a key source of chemokines, MMPs, and other inflammatory mediators that drive the initial cellular response [145]. Depletion of macrophages at this stage, although attenuating inflammation, also impairs wound debridement and compromises subsequent repair and regeneration [146]. As acute injury resolves, the initial inflammatory response subsides, and the macrophage population shifts toward an anti‐inflammatory, pro‐repair phenotype. These macrophages secrete abundant growth factors, including platelet‐derived growth factor, TGF‐β, insulin‐like growth factor 1, and vascular endothelial growth factor A, thereby promoting cell proliferation and angiogenesis. Concurrently, they release soluble mediators that stimulate fibroblast‐to‐myofibroblast differentiation, driving wound contraction, closure, and extracellular matrix synthesis [147]. Among these factors, TGF‐β stands out as the quintessential pro‐repair cytokine, driving fibroblast‐to‐myofibroblast polarization and enhancing matrix deposition and tissue remodeling. When the wound healing response proceeds in an orderly and controlled manner, the inflammatory response rapidly subsides, and normal tissue architecture is restored. However, disruption at any step of the repair process can result in aberrant healing, manifesting as either uncontrolled release of inflammatory mediators and growth factors or insufficient reparative macrophage function, ultimately leading to chronic wounds and pathological fibrosis. For example, macrophage depletion during the early phase of skin wound repair in mice impairs re‐epithelialization and vascularization, whereas depletion at later stages promotes fibrosis of the skin and liver [148].

In summary, macrophage function during tissue repair and remodeling evolves dynamically, exquisitely shaped by the postinjury temporal course, developmental origin, and microenvironmental factors such as tissue type and the nature and severity of injury. As master regulators of tissue repair, macrophages play indispensable roles at every stage—initiation, maintenance, and resolution—of this process [149]. Tissue remodeling and repair thus represent a multistage, sequentially orchestrated biological process mediated by macrophages. Its ultimate goal extends beyond restoring structural integrity to reestablishing the functional homeostasis of damaged tissues, thereby laying the foundation for the preservation of systemic homeostasis.

### 3.2 Key Signaling Pathways and Processes

Despite the remarkable heterogeneity of macrophages in developmental origin and functional repertoire, their functional fate converges upon a shared set of core signaling pathways. The dynamic balance of these pathways governs macrophage activation, effector functions, and polarization trajectories, and dissecting their molecular underpinnings has been a central endeavor in the field over the past decade. Under physiological conditions, precise regulation of these signaling networks ensures that macrophage functions remain appropriately calibrated and well‐ordered. However, when confronted with persistent or extreme stimuli, these regulatory networks can become dysregulated, precipitating pathological states such as chronic inflammation, fibrosis, and tumorigenesis. A deeper understanding of the regulatory logic operating across distinct macrophage functional states, therefore, not only illuminates the mechanisms that maintain homeostasis but also opens new therapeutic avenues for treating diverse diseases. In recent years, the widespread adoption of technologies such as live‐cell microscopy, high‐throughput single‐cell transcriptomics, and multiparametric flow cytometry has accelerated progress in bridging the gap between signaling pathways and functional outputs. Elucidating the specific molecular mechanisms by which macrophage dysfunction drives disease pathogenesis and leveraging this knowledge to design interventions that reverse pathological phenotypes now constitutes one of the most dynamic frontiers in the field.

#### 3.2.1 Endogenous Clearance and Recycling Signals

The clearance and recycling of endogenous aberrant materials and senescent or apoptotic cells by macrophages are governed by a precisely regulated signaling network. Chief among these clearance programs is efferocytosis, the programmed removal of apoptotic cells, which represents the most central and mechanistically well‐characterized process. This section, therefore, focuses on the key signaling pathways underlying endogenous clearance and recycling.

During apoptosis, plasma membrane integrity is preserved, and apoptotic cells release a repertoire of soluble chemoattractants into the surrounding microenvironment to recruit macrophages and enhance their clearance capacity. These “Find‐Me” signals display remarkable molecular diversity, encompassing modified membrane lipids such as lysophosphatidylcholine (LPC) and sphingosine‐1‐phosphate (S1P), nucleotides including ATP and uridine triphosphate (UTP), and chemokines such as fractalkine (encoded by CX3CL1) [150]. Among these, LPC and S1P represent apoptosis‐specific chemotactic signals. During apoptosis, caspase‐3 cleaves and activates calcium‐independent phospholipase A2, which then synthesizes LPC from phosphatidylcholine; Concurrently, a subset of apoptotic cells upregulates sphingosine kinases SPK1 and SPK2, which phosphorylate membrane sphingosine to generate S1P [151]. These signals establish chemotactic gradients that direct the migration of phagocytes toward apoptotic cells, ensuring the efficient initiation of clearance. As apoptosis progresses, the cell surface exposes “Eat‐Me” signals to trigger the phagocytic program. Phosphatidylserine, ordinarily confined to the inner leaflet of the plasma membrane in healthy cells, is externalized to the outer leaflet during apoptosis and represents one of the most critical signals [152]. Macrophages directly recognize these signals through a diverse array of phagocytic receptors, including complement receptors 3 and 4, T cell immunoglobulin and mucin domain‐containing proteins, the mannose receptor, CD36, scavenger receptors A/B, and integrins αvβ3 and αvβ5 [153]. Calreticulin on the apoptotic cell surface further facilitates phagocytosis by engaging low‐density lipoprotein receptor‐related protein 1. In contrast to the immunologically silent nature of apoptosis, nonapoptotic cell death is accompanied by loss of plasma membrane integrity, directly exposing surrounding cells to released inflammatory contents. In this context, damage‐associated molecular patterns derived from host cells are passively or actively liberated, acting both as chemoattractants that guide phagocyte migration to injury sites and as triggers of downstream inflammatory responses.

Notably, the engulfment of apoptotic cells can nearly double the lipid burden of macrophages, thereby triggering a metabolic adaptive transcriptional program centered on liver X receptor (LXR) and peroxisome proliferator‐activated receptor (PPAR) [154]. LXR alleviates lipid stress by promoting cholesterol efflux and fatty acid oxidation, whereas PPAR enhances sustained phagocytic capacity; together, they ensure the coordinated integration of metabolic homeostasis and phagocytic function during endogenous clearance. Furthermore, the initiation of phagocytosis is subject to negative regulation by “Don't‐Eat‐Me” signals, exemplified by the CD47‐signal regulatory protein alpha (SIRPα) axis [155], whose role in maintaining self‐immune tolerance will be elaborated later in this review. Beyond efferocytosis‐mediated clearance of apoptotic cells, the elimination of other endogenous waste materials by macrophages largely depends on direct recognition and internalization mediated by scavenger receptor family members such as scavenger receptor A (SR‐A) and CD36, as well as complement receptors [156], typically without engaging active chemotactic recruitment programs. The degradation of these substrates similarly proceeds through the lysosomal pathway; however, the upstream sensing signals and their coupling mechanisms with metabolic adaptation remain to be fully elucidated.

#### 3.2.2 Immune Surveillance and Tolerance Signals

Macrophages serve a dual role as both tissue‐resident sentinels and immune effector cells, their immune surveillance function playing an indispensable role in defending against exogenous threats such as viral infections. While earlier studies focused primarily on phagocytic clearance and antigen presentation, recent evidence demonstrates that macrophages in distinct activation states engage differential signaling pathways to exert bidirectional control over host immune responses. The dynamic shift of polarization states, coordinated with precise downstream signaling networks, directly shapes disease initiation and progression.

The initiation of immune surveillance depends on macrophage recognition of diverse danger signals, including PAMPs carried by invading microorganisms and IFN‐γ secreted by T helper 1 (Th1) cells. PAMPs are detected by macrophage pattern recognition receptors (PRRs), such as Toll‐like receptors (TLRs), the receptor for advanced glycation end products (RAGE), and Nod‐like receptors (NLRs). When cells infected with intracellular pathogens undergo non‐apoptotic death, such as pyroptosis, these pathogen‐specific molecules are released into the surrounding microenvironment upon cell rupture. Unlike host‐derived damage‐associated molecular patterns (DAMPs), PAMPs are conserved molecular structures unique to exogenous microorganisms and are absent from normal host cells. PAMP‐PRR engagement triggers downstream signaling cascades that promote the production of reactive oxygen and nitrogen intermediates, chemokines, pro‐inflammatory cytokines, and antimicrobial peptides, thereby enhancing macrophage phagocytic activity and effective clearance of microbial infections. Concurrently, IFN‐γ, the prototypical pro‐inflammatory cytokine, drives macrophage polarization toward an M1 phenotype, potentiating their immune surveillance capacity for eliminating intracellular pathogens [157]. Type I interferons upregulate IL‐10 production by macrophages to facilitate inflammation resolution, whereas type II interferon enhances MHC class II molecule expression to augment antigen presentation efficiency and promotes the secretion of inflammatory cytokines, nitric oxide, and reactive oxygen species to strengthen microbial clearance. Classically activated M1 macrophages present processed exogenous antigenic peptides on their surface for T cell receptor recognition, thereby initiating adaptive immune responses. However, when the inflammatory response becomes excessive, macrophages switch toward an M2 phenotype through inhibitory signals to counterbalance hyperinflammation and promote tissue repair. Alternatively activated M2 macrophages suppress inflammation by secreting anti‐inflammatory cytokines and expressing specific surface receptors, thus negatively regulating M1‐mediated immune surveillance; the underlying mechanisms will be detailed later in this review. Thus, the precise interplay between pro‐inflammatory and anti‐inflammatory signals during immune surveillance dictates the ultimate outcome of the immune response.

During immune surveillance, macrophage functional phenotypes are governed by a precisely orchestrated transcriptional signaling network, in which signal transducer and activator of transcription (STAT) family members serve as the core transcription factors mediating the M1/M2 polarization decision. IFN‐γ binds to its receptor and activates Janus kinase (JAK) kinases, which subsequently phosphorylate STAT1. Phosphorylated STAT1 forms homodimers that translocate to the nucleus and initiate the transcription of interferon‐stimulated genes, promoting M1 polarization and thereby enhancing pro‐inflammatory responses and pathogen clearance capacity. Additionally, upon Notch‐ligand interaction, the Notch intracellular domain (NICD) is proteolytically released and translocates to the nucleus, where it forms a transcriptional complex with CBF1/SU(H)/LAG‐1 (CSL) and Mastermind‐like transcriptional coactivators (MAML), driving the expression of pro‐inflammatory cytokines and M1‐associated genes. Notch activation biases macrophages toward M1 polarization to amplify inflammatory responses, whereas inhibition of this pathway promotes a shift toward an M2 phenotype, limiting inflammatory injury. Interleukin‐1 (IL‐1) further promotes M1 polarization through activation of the c‐Jun N‐terminal kinase (JNK) signaling pathway, constituting an additional branch of polarization control. These transcriptional regulatory networks engage in extensive crosstalk with PRR signaling. LPS, for instance, simultaneously triggers both myeloid differentiation primary response 88 (MyD88)‐dependent and MyD88‐independent pathways through Toll‐like receptor 4 (TLR4): the former primarily activates nuclear factor‐κB (NF‐κB) to promote the expression of inflammatory cytokines such as interleukin‐1β (IL‐1β), IL‐6, and tumor necrosis factor‐α (TNF‐α), whereas the latter activates interferon regulatory factor 3 (IRF3) to induce type I interferon production. Notably, Notch1 signaling can fine‐tune TLR4 signaling independently of MyD88, with LPS further potentiating TLR4 activation through the Notch1 pathway, synergistically driving macrophages toward the M1 phenotype and enhancing cytokine secretion. While these pathways drive immune surveillance forward, the maintenance of immune tolerance depends on negative regulation of phagocytic activity by “Don't‐Eat‐Me” signals. CD47 on the erythrocyte surface engages SIRPα (CD172a) to deliver a self‐signal that inhibits phagocytosis, and the rapid clearance of erythrocytes in CD47‐deficient mice validates the essential role of this pathway in maintaining self‐tolerance. Similarly, the interaction between CD200 and CD200R transmits an inhibitory signal to macrophages, restraining their activation.

In summary, the delicate balance between immune surveillance and immune tolerance relies on the coordinated regulation of signaling pathways, transcriptional networks, and “Don't‐Eat‐Me” signals, which collectively determine the functional orientation of macrophages in defending against exogenous threats while preserving self‐tolerance.

#### 3.2.3 Tissue Remodeling and Repair Signals

The functional transition of macrophages during tissue repair is governed by a multilayered and precisely orchestrated signaling network. The TGF‐β pathway, the best‐characterized core mechanism, drives fibroblast‐to‐myofibroblast polarization through both Smad‐dependent and Smad‐independent cascades, enhancing extracellular matrix deposition and promoting tissue remodeling, thereby constituting a critical regulatory node in reparative fibrosis [158].

The efferocytosis‐associated signaling network serves as the central hub linking apoptotic cell clearance to the acquisition of a reparative phenotype. Externalized phosphatidylserine on the apoptotic cell surface and released intracellular molecules, such as ATP, recruit opsonins, including milk fat globule‐EGF factor 8 protein (Mfge8) and annexin A1 (AnxA1), and promote the binding of protein S and growth arrest‐specific 6 (GAS6) to phosphatidylserine [159]. Protein S and GAS6 function as ligands for the TAM receptor tyrosine kinase family (Tyro3, Axl, Mer), with bridging molecules such as T‐cell immunoglobulin and mucin domain containing 4 (TIM4) and DEL‐1 facilitating their engagement with TAM receptors [160]. Activation of TAM receptors engages a downstream transcriptional network that includes LXR‐α, LXR‐β, and PPAR‐γ, thereby suppressing inflammatory signaling and promoting the transition toward a reparative phenotype. In microglia, triggering receptor expressed on myeloid cells 2 (TREM2) sustains efferocytic capacity by binding phospholipids on the apoptotic cell surface [161]. Its deficiency impairs efferocytosis and upregulates inflammatory gene transcription. Efferocytosis can also induce the proliferation of reparative macrophages through Mer‐dependent downstream signaling and the phagolysosomal degradation pathway.

Cytokine signaling networks constitute the direct regulatory layer that drives reparative polarization. IL‐4 and IL‐13, through activation of the STAT6 signaling axis, cooperate with apoptotic cell‐sensing signals to promote macrophage transition toward a reparative phenotype. IL‐33, a highly conserved alarmin released upon tissue injury, promotes reparative macrophage polarization [162]. Macrophage‐derived IL‐10 suppresses inflammatory responses in an autocrine manner, in part by promoting autophagy to clear dysfunctional mitochondria, thereby restricting NLR family pyrin domain containing 3 (NLRP3) inflammasome activation and establishing an immune microenvironment permissive for orderly repair [163].

In addition, the phosphoinositide 3‐kinase (PI3K) and protein kinase B (Akt) signaling pathways integrate diverse upstream signals to promote macrophage polarization toward a reparative phenotype during tissue repair [164]. The JNK, a member of the mitogen‐activated protein kinase (MAPK) family, displays dual regulatory functions, driving pro‐inflammatory responses under certain conditions while facilitating reparative polarization under others [165]. Regulatory T cells can directly modulate macrophage reparative functions through the release of amphiregulin (AREG), TGF‐β, and IL‐10, or via interactions mediated by CD40 and CD80. MMPs, secreted as key effector molecules by macrophages, degrade various extracellular matrix components, among which MMP12 is highly induced by IL‐13 in the lung and liver and contributes to fibrotic progression [166]. At the metabolic level, pro‐resolving lipid mediators synthesized by macrophages, including resolvins, protectins, and maresins [167], actively promote inflammation resolution and create favorable microenvironmental conditions for tissue repair, while the endogenous metabolite itaconate mitigates secondary damage to tissue architecture by suppressing excessive inflammatory responses.

In summary, the functional transition of macrophages during tissue repair is coordinated by a multilayered regulatory network comprising the core TGF‐β pathway, the efferocytosis hub, direct cytokine‐mediated regulation, and auxiliary signals including PI3K/Akt and JNK. This integrated control ensures the orderly progression of repair programs and the ultimate reestablishment of tissue homeostasis.

## 4 Macrophages in Human Diseases: A Double‐Edged Sword

**FIGURE 3:**
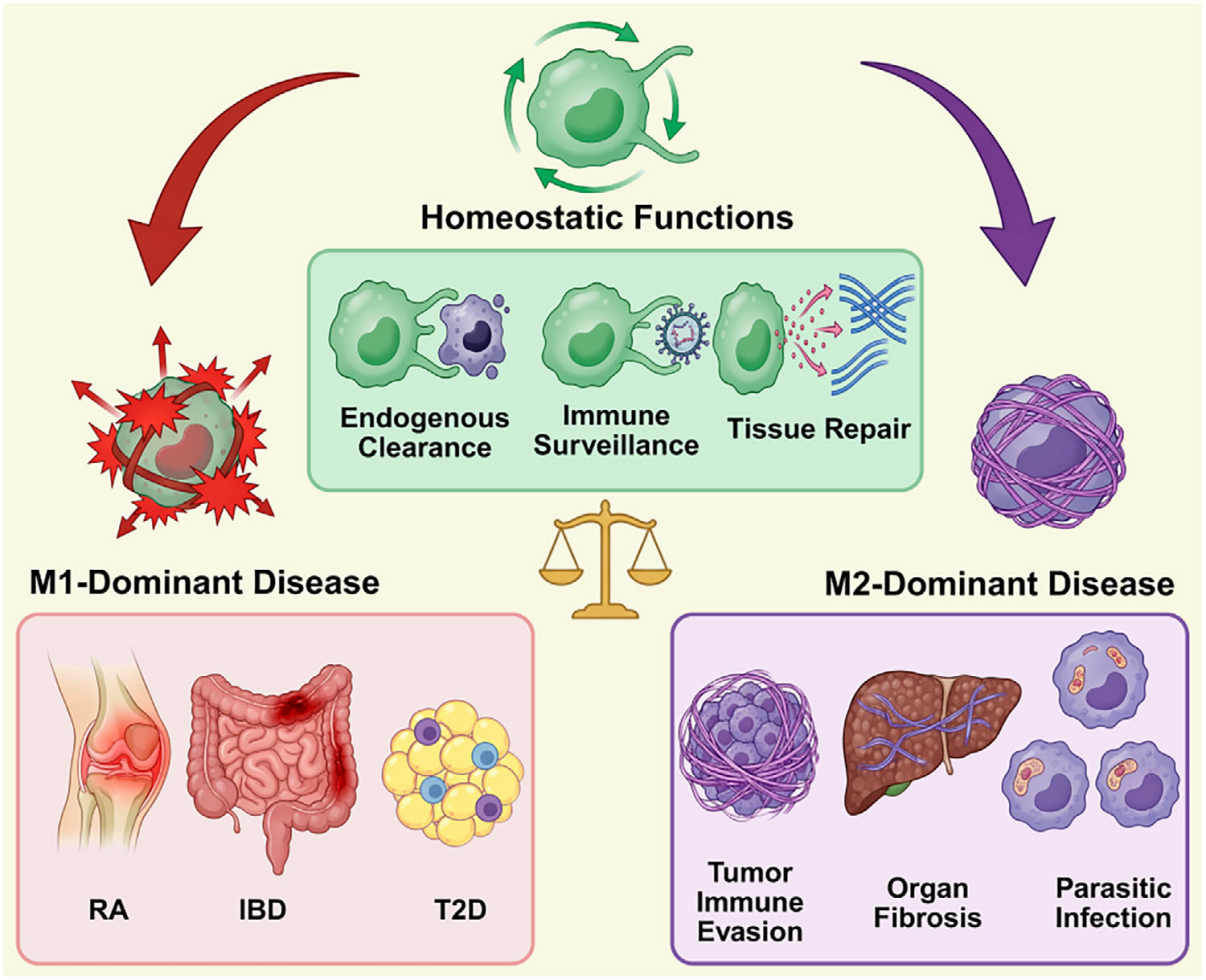
M1/M2 polarization imbalance shifts macrophages from homeostatic guardians to drivers of disease. Under homeostatic conditions, macrophages maintain tissue integrity and organismal homeostasis through three core physiological functions: endogenous clearance, immune surveillance, and tissue repair. Disruption of the M1/M2 balance skews macrophages toward either M1‐dominant or M2‐dominant functional states. M1‐skewed polarization drives chronic inflammation and autoimmunity, including rheumatoid arthritis, inflammatory bowel disease, and type 2 diabetes. M2‐skewed polarization facilitates tumor immune evasion, organ fibrosis, and persistent parasitic infections. (Created in https://BioRender.com).

The functional properties of macrophages are not rigidly predetermined by their developmental origin but are instead dynamically shaped and continuously calibrated by the signaling networks within the tissue microenvironment. This remarkable plasticity enables the same macrophage population to adopt strikingly different functional phenotypes depending on the disease stage or signaling context. Thus, macrophages are not intrinsically “good” or “bad”; rather, whether they exert protective or pathogenic roles depends entirely on the congruence between their activation state and the specific pathological setting. When the precise coordination between microenvironmental signals and macrophage responses becomes disrupted, homeostatic guardians are transformed into disease drivers (Figure **3**) [168]. At the molecular level, this dysregulation manifests predominantly as a breakdown of the M1/M2 polarization balance, and the pathogenesis of numerous diseases is intimately linked to such an imbalance. Polarization imbalance represents both a consequence of disordered microenvironmental signals and a driving force that sustains disease progression. Consequently, macrophage‐targeted therapeutic strategies should extend beyond the mere elimination of specific subsets and instead center on correcting polarization imbalance as the primary point of intervention. The central objective is to modulate macrophage polarization states to suppress pathogenic functions while promoting protective ones, thereby restoring the dynamic equilibrium between M1 and M2 and reestablishing immune homeostasis within the tissue microenvironment.

### 4.1 Cancer: The Prototypic Role of TAMs

The persistent inflammatory state within the tumor microenvironment is now recognized as a hallmark of cancer, and macrophages, as the most abundant immune cells in this milieu, engage in complex interactions with tumor cells that profoundly influence tumor evolution. These TAMs are key drivers of tumor progression, metastasis, and therapeutic resistance and constitute the dominant immune infiltrate within the tumor microenvironment [169]. TAMs arise predominantly from circulating monocytes recruited by tumor‐secreted chemokines, with a minor contribution from tissue‐resident macrophages. In glioblastoma, for instance, bone marrow‐derived macrophages account for over 85% of total TAMs, whereas resident microglia represent only approximately 15% [170].

The signaling networks within the tumor microenvironment profoundly shape TAM heterogeneity and functional orientation, endowing them with dual roles during tumor progression. Upon recruitment, monocyte‐derived TAMs polarize under local stimulation into either an antitumor M1‐like phenotype or a pro‐tumor M2‐like phenotype, acquiring corresponding functional properties. Under the influence of IFN‐γ, TNF‐α, and granulocyte‐macrophage colony‐stimulating factor (GM‐CSF), M1‐like TAMs exhibit enhanced antigen‐presenting capacity, activate Th1‐type immune responses, and directly eliminate tumor cells, thereby exerting tumor‐suppressive effects [171]. Conversely, M2‐like TAMs, generated in the presence of IL‐10 and TGF‐β, activate Th2‐type responses and promote tumor initiation and progression. Within the tumor microenvironment, M2‐like TAMs drive tumor progression and are associated with poor prognosis through multiple mechanisms, including promoting angiogenesis, mediating immunosuppression, and facilitating metastasis. M2‐like TAMs secrete an array of growth factors such as vascular endothelial growth factor, platelet‐derived growth factor, epidermal growth factor, and TGF‐β, while simultaneously releasing matrix metalloproteinases that remodel the extracellular matrix to facilitate neovascularization. For example, in ovarian cancer, TAM‐derived epidermal growth factor activates tumor cell EGFR signaling and upregulates the VEGF/VEGFR pathway, cooperatively driving vascular network formation [172]. Furthermore, M2‐like TAMs suppress the activation and proliferation of T cells and natural killer cells through the secretion of IL‐10 and TGF‐β, and can directly impair T cell cytotoxic function via the PD‐L1/PD‐1 axis. In non–small cell lung cancer, M2‐like TAMs expressing the macrophage collagen receptor MARCO block the activation of cytotoxic T cells and natural killer cells, inhibit their proliferation and cytokine production, and reshape the immunosuppressive microenvironment by expanding regulatory T cells [173]. Concurrently, M2‐like TAMs secrete C‐X‐C motif chemokine ligand 8 (CXCL8) and TGF‐β, which enhance tumor cell migration and invasion and induce epithelial–mesenchymal transition. In bladder cancer, TAM infiltration‐driven CXCL8 elevation promotes tumor cell secretion of matrix metalloproteinase‐9 (MMP‐9) and downregulation of E‐cadherin, thereby significantly enhancing migration and invasion [174]. Recent studies have also revealed that TAMs can promote intratumoral lymphangiogenesis, further expanding their pro‐tumorigenic functional repertoire.

Critically, TAMs can reversibly respond to specific stimuli within the tumor microenvironment and dynamically transition between antitumor M1‐like and pro‐tumor M2‐like phenotypes under appropriate immunological conditions [175, 176]. This plasticity provides the conceptual foundation for TAM functional reprogramming as an antitumor strategy, wherein directed modulation of their polarization state can restore antitumor defense functions [177]. In recent years, TAM functional plasticity has been further validated in preclinical models and clinical trials, and strategies aimed at targeted polarization control have shown early signs of enhanced antitumor immunity. The phosphoinositide 3‐kinase gamma (PI3K‐γ) pathway and the colony‐stimulating factor‐1/colony‐stimulating factor‐1 receptor (CSF‐1/CSF‐1R) signaling axis have been demonstrated to play critical roles in M2‐like TAM polarization [178]. In a pancreatic ductal adenocarcinoma model, dual blockade of PI3K‐γ and CSF‐1/CSF‐1R drove the transition of TAMs from an M2‐like to an M1‐like state, reducing the number of immunosuppressive macrophages and enhancing CD8^+^ T cell responses. Inhibition of CSF‐1/CSF‐1R alone has achieved similar effects in models of glioblastoma, melanoma, and rhabdomyosarcoma. Furthermore, signal transducer and activator of transcription 3 (STAT3) and STAT6 govern pro‐tumor macrophage polarization; STAT3 inhibitors markedly reduce M2‐like polarization in glioma, while TAMs from STAT6‐deficient mice display an M1‐like phenotype accompanied by enhanced antitumor immunity [179].

Inhibiting the pro‐tumorigenic functions of TAMs can effectively enhance the antitumor activity of the tumor microenvironment and reverse its immunosuppressive state. However, the remarkable heterogeneity of TAMs—with antitumor M1‐like and pro‐tumor M2‐like subsets coexisting and exerting opposing effects on tumor progression—directly impacts the design of current TAM‐targeted immunotherapeutic strategies. Deeper characterization of specific markers for distinct functional TAM subsets will therefore provide a critical foundation for the development of more precise targeted interventions.

### 4.2 Autoimmune and Inflammatory Diseases

Under physiological conditions, macrophages maintain a dynamic equilibrium between M1 pro‐inflammatory and M2 anti‐inflammatory phenotypes to ensure the precision and appropriate magnitude of immune responses. When this balance is disrupted, and macrophages become persistently locked into a pro‐inflammatory M1 state, their excessive release of inflammatory cytokines, reactive oxygen species, and matrix metalloproteinases triggers tissue damage, breaks immune tolerance, and drives chronic inflammation [180, 181]. This M1‐skewed polarization can be reinforced by aberrant activation of multiple signaling pathways, including Notch, JAK/STAT, NF‐κB, and MAPK cascades, and is coupled with metabolic reprogramming—particularly enhanced glycolysis—that perpetuates the pro‐inflammatory phenotype. The consequent disruption of immune homeostasis can affect multiple tissues and organs, such as the synovium, intestine, kidneys, and skin, underpinning a spectrum of autoimmune and inflammatory diseases. Among these, rheumatoid arthritis and inflammatory bowel disease represent prototypical conditions driven by M1/M2 polarization imbalance: the former is characterized by persistent synovial inflammation and bone erosion mediated by synovial macrophages, whereas the latter involves mucosal barrier disruption and chronic relapsing inflammation driven by intestinal macrophage dysfunction. This section will systematically delineate the pathogenic mechanisms of macrophage polarization imbalance and the corresponding therapeutic strategies, using these two diseases as representative examples.

Rheumatoid arthritis is a chronic autoimmune disease characterized by persistent synovial inflammation, cartilage erosion, and progressive joint destruction. As central effector cells, macrophages disrupt immune homeostasis and amplify joint inflammation through M1/M2 polarization imbalance [182, 183]. Synovial macrophage abundance correlates positively with joint damage severity: M1 macrophages predominate in active disease, whereas M2 macrophages are associated with clinical remission [184]. In early disease, pro‐inflammatory mediators released by M1 macrophages promote further monocyte recruitment and activation, amplifying synovial inflammation. M1/M2 imbalance also disrupts bone homeostasis, leading to aberrant bone remodeling and bone loss. As the disease progresses, neovascularization, synovial lining hyperplasia, and pannus formation collectively drive cartilage destruction, with inflammatory macrophages contributing to articular surface erosion through matrix metalloproteinase production. Thus, M1 macrophages, through their pro‐inflammatory and tissue‐destructive activities, are linked to more severe disease progression, whereas M2 macrophages promote inflammation resolution by clearing apoptotic cells and debris and producing anti‐inflammatory mediators. Reprogramming macrophages from an M1 to an M2 phenotype may therefore alleviate inflammatory damage and suppress bone destruction. These functional differences are closely linked to the subset composition of synovial macrophages. In human synovium, tissue‐resident and infiltrating subsets can be distinguished by MerTK and CD206 expression. Tissue‐resident macrophages constitute the predominant population in healthy synovium and comprise a TREM2^+^CX3CR1^+^FOLR2^+^ subset forming a protective lining on the synovial surface and a LYVE1^+^FOLR2‐high subset in the sublining layer [185]. During arthritis, tissue‐resident macrophages further upregulate CD163 expression. Precise targeting of specific synovial macrophage subsets for phenotypic reprogramming thus represents a promising therapeutic strategy. Multiple signaling pathways, including Notch, ERK, JAK/STAT, and MAPK, regulate M1 polarization. Notch signaling is activated in the inflamed joint, and its inhibition promotes an M1‐to‐M2 shift, reducing inflammation and bone loss. Similarly, interferon‐γ activates the JAK/STAT1 pathway to drive pro‐inflammatory mediator release from M1 macrophages, and STAT1 inhibition can promote an M2 shift.

Inflammatory bowel disease, encompassing Crohn's disease and ulcerative colitis, comprises a group of chronic, relapsing inflammatory disorders primarily affecting the gastrointestinal tract [186, 187, 188]. The intestine harbors the largest macrophage population in the body. Under physiological conditions, lamina propria macrophages predominantly display an M2 phenotype, characterized by low expression of pro‐inflammatory cytokines and chemokines and high expression of phagocytic receptors. These macrophages, through their highly active phagocytic capacity, continuously clear cellular debris, apoptotic cells, and harmless commensal bacteria in an immunologically silent manner, thereby maintaining intestinal immune homeostasis. As disease progresses, however, the dynamic M1/M2 balance is disrupted, and the resulting polarization imbalance becomes a key driver of sustained intestinal inflammation. Upon barrier disruption, macrophages skew toward a pro‐inflammatory M1 phenotype, secreting large quantities of inflammatory mediators that exacerbate epithelial damage, promote apoptosis, and stimulate granulation tissue formation. In patients, a marked increase in M1 macrophages is accompanied by a reduction in M2 macrophage numbers. The loss of M2‐mediated anti‐inflammatory and reparative functions impairs the clearance of apoptotic cells and pathogens, further exacerbating antigen exposure and immune dysregulation and fueling a vicious cycle of inflammation. Enhancing M2 macrophage function while suppressing aberrant M1 responses, therefore, constitutes a core therapeutic strategy for these diseases [189].

In autoimmune and inflammatory diseases such as inflammatory bowel disease, single‐cell studies have identified multiple functional states of activated macrophages, including a CXCL5‐high M1 pro‐inflammatory subset, an aconitate decarboxylase 1 (ACOD1)‐high M1 metabolically activated subset, and inflammation‐dependent alternative macrophage clusters marked by neuregulin 1 (NRG1), AREG, and heparin‐binding EGF‐like growth factor (HBEGF). Under physiological conditions, CSF‐1 preferentially drives monocyte differentiation toward an M1 phenotype, whereas IL‐34 tends to induce an M2 phenotype. In disease states, however, both cytokines are highly expressed and exert pro‐inflammatory effects, and combined blockade significantly ameliorates disease symptoms [190]. Vitamin D receptor signaling improves colitis in mice by promoting M1‐to‐M2 conversion. PI3K/Akt pathway activation promotes M2 differentiation and anti‐inflammatory responses; accordingly, anti‐TNF therapy or AMPK activation can increase the M2 macrophage proportion and confer anti‐inflammatory effects [191]. Thus, although rheumatoid arthritis and inflammatory bowel disease affect distinct organs, both share M1/M2 macrophage polarization imbalance as a central pathological hub. Restoring the dynamic equilibrium between pro‐inflammatory and anti‐inflammatory phenotypes, therefore, constitutes a common conceptual foundation for the targeted therapy of autoimmune and inflammatory diseases.

### 4.3 Metabolic Diseases

Macrophages serve as central sensors and effectors within the metabolic regulatory network. Under physiological conditions, tissue‐resident macrophages predominantly display an M2 anti‐inflammatory phenotype and participate in the fine‐tuned maintenance of metabolic homeostasis. When this equilibrium is disrupted by factors such as nutrient excess, however, macrophages undergo a marked shift from an M2 toward an M1 phenotype. This polarization imbalance drives metabolic organs, including adipose tissue and the liver, into a state of chronic low‐grade inflammation, ultimately precipitating systemic insulin resistance. Consequently, macrophages transition from executors of metabolic homeostasis to drivers of metabolic dysregulation, with M1/M2 polarization imbalance constituting a common immunopathological foundation for a broad spectrum of metabolic diseases. Promoting M1‐to‐M2 reprogramming to restore polarization balance has therefore emerged as a promising therapeutic strategy. This section will systematically delineate the pathogenic mechanisms of macrophage polarization imbalance in metabolic diseases and the corresponding targeted intervention strategies, using obesity and its common complications, type 2 diabetes and nonalcoholic fatty liver disease, as representative examples.

Macrophages represent the most abundant immune cell population in adipose tissue. In the obese microenvironment, the recruitment of circulating monocytes to adipose tissue is markedly enhanced, and the local proliferation of tissue‐resident macrophages is also significantly increased. This dual drive elevates the proportion of macrophages within the stromal vascular fraction from approximately 10% in the lean state to roughly 50% in obesity [192, 193]. This quantitative expansion is accompanied by profound phenotypic and functional alterations. Nutrient excess and obesity activate a cascade of metabolic disturbances, including inflammatory signaling, lipotoxicity, adipokine dysregulation, hypoxia, endoplasmic reticulum stress, and mitochondrial dysfunction. These pathways collectively drive the polarization of adipose tissue macrophages toward a pro‐inflammatory M1 phenotype and precipitate their functional dysregulation. This metabolic reprogramming is characterized by sustained glycolytic flux and a truncation of the TCA cycle at SDH, leading to succinate accumulation and subsequent HIF‐1α‐dependent transcription of IL‐1β. Consequently, these macrophages emerge as central executors of metabolic inflammation, ultimately driving both local and systemic insulin resistance. Adipose tissue macrophages constitute a highly heterogeneous population that exists along a continuous phenotypic spectrum extending from anti‐inflammatory to pro‐inflammatory states [194, 195]. Under physiological conditions, M2 macrophages, characterized by their anti‐inflammatory properties, constitute the resident macrophage population in lean adipose tissue [196]. These cells characteristically express surface markers such as CD11b, F4/80, CD301, and CD206, and maintain normal insulin signaling through the secretion of anti‐inflammatory cytokines, including IL‐10. In obesity, however, M1 macrophages are recruited in large numbers to white adipose tissue, where they secrete inflammatory mediators such as TNF‐α, IL‐1β, leukotriene B4, and nitric oxide, thereby impairing normal insulin signal transduction in adipocytes [197]. With the sustained accumulation of M1 macrophages, the M1‐to‐M2 ratio increases substantially, and M1/M2 polarization imbalance thus becomes a critical nodal point in disease progression.

The key distinction between metabolically healthy and metabolically unhealthy obesity lies in the trajectory of this polarization balance. Metabolically healthy obese individuals maintain a higher M2/M1 ratio [198]. Their adipocytes secrete type 2 cytokines such as IL‐4 and IL‐13, which activate PPARδ, thereby promoting M2 polarization and enhancing systemic insulin sensitivity. PPARγ is expressed at higher levels in insulin‐sensitive obese individuals and drives healthy adipocyte proliferation in response to nutrient excess. Within this virtuous cycle, M2 macrophages support preadipocyte survival and differentiation, generating numerous smaller adipocytes that help consolidate an anti‐inflammatory microenvironment. In contrast, in metabolically unhealthy obese individuals, hypertrophic adipocytes recruit M1 macrophages and secrete pro‐inflammatory mediators such as leukotriene B4, establishing a vicious cycle of escalating M1 infiltration, impaired adipogenic differentiation, and worsening insulin resistance. This polarization imbalance is under the precise control of local microenvironmental signals. In lean adipose tissue, high levels of IL‐4 maintain the M2 phenotype through the induction of PPARγ and PPARδ expression, whereas saturated fatty acids and interferon‐γ drive M1 polarization in obesity. Furthermore, healthy adipose tissue expansion depends on adaptive remodeling of the extracellular matrix and adequate angiogenesis. Under physiological conditions, M2 macrophages promote matrix metalloproteinase activation by downregulating TIMP‐1, thereby maintaining efficient matrix turnover and driving neovascularization. In metabolically unhealthy obesity, however, excessive collagen deposition leads to interstitial fibrosis, accompanied by elevated MMP‐9 and MMP‐14 expression and capillary rarefaction. Declining VEGF signaling further exacerbates tissue hypoxia and drives the progression of insulin resistance.

Obesity‐induced systemic low‐grade inflammation establishes a shared immunopathological foundation for both type 2 diabetes and nonalcoholic fatty liver disease. In type 2 diabetes, M1 polarization skewing occurs in macrophages across multiple organs, including adipose tissue, skeletal muscle, pancreatic islets, and the liver, cooperatively driving systemic insulin resistance and hyperglycemia [199, 200]. In nonalcoholic fatty liver disease, hepatic macrophage M1 polarization and foam cell formation dominate the processes of hepatic lipid accumulation, inflammatory cascades, and disease progression. Although these conditions affect distinct organs and present with different clinical manifestations, both are fundamentally anchored by M1/M2 polarization imbalance as a central hub, accompanied by the coordinated dysregulation of multilayered regulatory networks involving metabolic reprogramming and signaling pathway hijacking. In adipose tissue, M1 macrophages secrete TNF‐α, IL‐1β, and IL‐6, which activate the JNK and NF‐κB pathways to impair insulin signaling and suppress GLUT4 translocation to the plasma membrane. This succinate–HIF‐1α–IL‐1β cascade thereby establishes a direct mechanistic link between immunometabolic reprogramming and adipocyte insulin resistance. Interstitial M1 macrophages in skeletal muscle impair myocyte glucose uptake through the paracrine actions of IL‐1β and TNF‐α. In pancreatic islets, sustained hyperglycemia and elevated free fatty acids drive M1 macrophage activation, and the IL‐1β released by these cells suppresses β‐cell insulin gene expression and induces apoptosis via the NF‐κB pathway. Clinical studies have demonstrated that the number of CD68‐positive macrophages in the islets of patients with type 2 diabetes is significantly higher than that in nondiabetic controls, and that inflammation precedes β‐cell apoptosis. IL‐1β signaling exhibits a threshold effect: transient low‐concentration exposure can promote insulin secretion, whereas sustained exposure triggers irreversible β‐cell dysfunction [201]. In the liver, lipotoxicity drives the massive recruitment of monocyte‐derived macrophages, which surpass Kupffer cells to become the dominant macrophage population, and their release of pro‐inflammatory mediators exacerbates hepatic insulin resistance. Concurrently, macrophage scavenger receptor MSR1 is upregulated in nonalcoholic steatohepatitis, where it mediates the unrestricted uptake of modified lipoproteins and promotes foam cell formation, further amplifying hepatic lipid deposition and the inflammatory cascade [202]. MSR1‐deficient mice fed a high‐fat, high‐sugar diet in a model of nonalcoholic steatohepatitis exhibit reduced hepatic foam cell formation, attenuated inflammation, and improved glucose tolerance.

In summary, therapeutic strategies targeting macrophage polarization balance have achieved preliminary validation in metabolic diseases. In obesity, PPARγ agonists promote the healthy expansion of subcutaneous adipose tissue and drive M2 polarization, improving insulin sensitivity while maintaining adipose tissue homeostasis [203], while type 2 cytokines such as IL‐4 and IL‐13 activate the PPARδ pathway to synergistically enhance M2 polarization. In type 2 diabetes and nonalcoholic fatty liver disease, metformin suppresses M1 polarization and attenuates hepatic steatosis through AMPK activation, SGLT2 inhibitors inhibit M1 polarization while promoting an M2 shift, and GLP‐1 receptor agonists directly modulate macrophage function by inhibiting NF‐κB signaling, thereby improving insulin sensitivity and reducing hepatic steatosis. These strategies converge on a common goal: promoting M1‐to‐M2 reprogramming across multiple organs, including adipose tissue, liver, and pancreatic islets, to restore polarization balance, thereby offering a promising interventional avenue for treating obesity and its associated metabolic complications. Beyond conventional metabolic agents, future strategies that target immunometabolic nodes—such as interrupting succinate‐driven inflammatory signaling or potentiating itaconate‐mediated NRF2 activation—may achieve more durable restoration of immunometabolic homeostasis.

### 4.4 Fibrotic Diseases

Fibrosis is a pathological process characterized by excessive extracellular matrix deposition and essentially represents an aberrant wound healing response driven by chronic inflammation. Myofibroblasts, as the principal source of extracellular matrix, directly govern the progression and regression of fibrosis through their activation and expansion. Recent studies have further revealed that macrophages not only function as upstream immune regulators of myofibroblasts but also serve as an important source of myofibroblasts themselves. Under specific signaling cues, macrophages can directly transdifferentiate into myofibroblasts, a process termed macrophage‐to‐myofibroblast transition (MMT) [204, 205]. Cells undergoing MMT co‐express the macrophage marker CD68 and the myofibroblast marker α‐smooth muscle actin, and this process is precisely regulated by the transforming growth factor‐β1/Smad3 signaling axis. However, although MMT has been proposed to play a potential role in fibrosis, lineage tracing studies have yielded conflicting results regarding its in vivo extent and functional significance, and more rigorous genetic evidence is required to clarify this issue [206]. The monocyte‐macrophage population exerts bidirectional regulatory functions at different stages of fibrosis, capable of driving both pro‐fibrotic and antifibrotic effects. Multiple reprogramming factors can disrupt the dynamic equilibrium between M1 and M2 phenotypes, promoting the skewing and switching of macrophages between pro‐inflammatory and pro‐fibrotic states [207]. This section examines liver fibrosis, pulmonary fibrosis, and kidney fibrosis as representative examples to systematically delineate the pathogenic mechanisms of macrophage polarization imbalance in fibrotic diseases and the corresponding targeted intervention strategies.

Under chronic liver injury, circulating monocytes are preferentially recruited to the inflamed liver, where they drive fibrogenesis through the secretion of pro‐inflammatory and pro‐fibrotic mediators [208]. Activated hepatic stellate cells further amplify macrophage infiltration and induce M2 polarization via the CCL2/CCR2 axis, establishing a positive feedback loop that exacerbates fibrosis. Kupffer cells, as the resident macrophage population of the liver, exert bidirectional regulation of fibrotic progression by modulating the M1/M2 polarization balance. M2‐skewed Kupffer cells can promote the apoptosis of M1 macrophages and play protective roles in alcohol‐ and high‐fat diet‐induced liver injury. Activation of the cannabinoid receptor CB2 drives an M1‐to‐M2 shift, and its antifibrotic effects are closely linked to the suppression of IL‐17‐mediated pro‐inflammatory signaling [209]. Furthermore, inhibition of the Notch signaling pathway reduces M1 polarization and promotes M2 skewing. When injurious signals subside, liver fibrosis enters a regression phase, during which activated hepatic stellate cells undergo programmed apoptosis, accompanied by an increased ratio of matrix metalloproteinases to their tissue inhibitors. Macrophage‐secreted TNF‐related apoptosis‐inducing ligand can mediate this process, and these macrophages are referred to as restorative macrophages. Mesenchymal stem cell transplantation exerts synergistic effects in reversing liver fibrosis by promoting M2 polarization while suppressing M1 polarization.

Under chronic lung injury, circulating monocytes are recruited to the inflamed lung tissue, where they drive fibrogenesis by secreting pro‐inflammatory and pro‐fibrotic mediators. These cells highly express CCR2, and activated alveolar epithelial cells promote macrophage infiltration and M2 polarization via the CCL2/CCR2 axis. M2 macrophages, characterized by CD206 and YM1 expression, are polarized by IL‐4 and IL‐13. IL‐10 overexpression amplifies Th2‐type responses through the induction of IL‐4 and IL‐13, whereas IL‐9 exerts antifibrotic effects by limiting type 2 polarization. Upon resolution of injury, serum amyloid P improves pulmonary fibrosis by suppressing M2 skewing, while depletion of Ly6C‐high monocytes can target M2 macrophages to attenuate disease progression [210]. Thus, both the development and regression of pulmonary fibrosis depend on dynamic shifts in macrophage polarization balance, and fine‐tuning this balance constitutes a core therapeutic strategy.

Similarly, macrophage polarization balance governs the development and regression of kidney fibrosis. Under chronic kidney injury, monocyte‐derived macrophages undergo M2 polarization. These M2 macrophages express CD206 and arginase‐1: the M2c subset suppresses early inflammation by inducing regulatory T cell infiltration, whereas persistently present M2 macrophages drive fibrotic progression through TGF‐β1 secretion. The renin–angiotensin system plays an important regulatory role in this process; angiotensin type 1 receptor blockers promote M1‐to‐M2 switching, which in turn facilitates epithelial–mesenchymal transition and aggravates fibrosis.

Therapeutic strategies targeting MMT have been preliminarily validated in multiorgan fibrosis models. Eplerenone inhibits MMT and attenuates fibrotic lesions by blocking the mineralocorticoid receptor pathway. Adenosine A2B receptor antagonists reduce macrophage infiltration and MMT occurrence within glomeruli. Quercetin mitigates pulmonary fibrosis by suppressing macrophage phenotypic switching and the TGF‐β/Smad2/3 pathway [211]. Collectively, these findings indicate that intervening at critical nodes of MMT to halt fibrotic progression represents a promising strategy for the treatment of fibrotic diseases.

### 4.5 Infectious Diseases

Infectious diseases arise from the invasion of pathogens, including bacteria, fungi, viruses, and parasites. Their onset and outcome depend not only on the virulence and invasion strategies of the pathogen but also, critically, on the strength and balance of the host–immune response. In human infectious diseases, the macrophage immune response does not adopt a fixed pro‐inflammatory or anti‐inflammatory state; rather, it operates as a dynamic continuum between these two functional poles. Macrophages play a dual role, serving both as host cells for pathogen residence and as effector cells that execute pathogen killing [212]. This section systematically delineates the pathogenic mechanisms of macrophage polarization imbalance in infectious diseases and the corresponding targeted intervention strategies, addressing bacterial, fungal, viral, and parasitic pathogens in turn.

Acute infection with *Mycobacterium tuberculosis* induces macrophage polarization toward an M1 phenotype and suppresses IL‐10 production through the nuclear receptor REV‐ERBα, thereby enhancing bactericidal activity and reducing intracellular pathogen survival. However, intracellular *M. tuberculosis* can also inhibit the transcription of interferon‐γ target genes to attenuate M1 polarization [213], thereby promoting the formation of tuberculous pleural effusions. As tuberculous granulomas form and progress, macrophage activation undergoes a dynamic transition from an M1‐like to an M2‐like state. Furthermore, diverse pathogens, including *Helicobacter pylori*, *Salmonella typhimurium*, *Salmonella typhi*, and *Candida albicans*, also drive macrophage polarization toward an M2‐like phenotype and suppress host inflammatory responses through distinct mechanisms.

Similar to bacterial pathogens, viruses and parasites have evolved a variety of strategies to hijack the macrophage polarization process. Upon pathogen recognition, macrophages phagocytose the invaders and polarize toward an M1‐like phenotype, presenting antigens via major histocompatibility complex molecules to T lymphocytes. The latter secrete interferon‐γ, which recruits effector immune cells and induces inflammatory responses to control pathogen replication. Human cytomegalovirus serves as a paradigm for the infection stage‐dependent dynamic regulation of polarization balance. During early infection, it induces infected monocytes to adopt a mixed polarization state biased toward an M1‐like phenotype, exploiting the M1‐driven pro‐inflammatory microenvironment to facilitate the migration and dissemination of infected cells to tissues. During late infection, however, it drives a shift of infected cells toward M2‐like polarization through viral IL‐10, thereby subverting host clearance. Similarly, in human post‐kala‐azar dermal leishmaniasis, patient macrophages exhibit M2‐like polarization skewing, characterized by reduced TLR‐2 and TLR‐4 expression and diminished nitric oxide production, accompanied by increased expression of CD206 and arginase‐1, together with elevated levels of IL‐4, IL‐10, and IL‐13 that favor persistent parasite survival [214].

In summary, although distinct pathogens manipulate macrophage polarization through diverse molecular mechanisms, their common strategy lies in subverting the dynamic equilibrium between pro‐inflammatory and anti‐inflammatory phenotypes to create conditions permissive for their own survival and dissemination. Precisely modulating the timing and direction of macrophage polarization to balance pathogen clearance with tissue protection, therefore, represents a promising central strategy for the treatment of infectious diseases.

### 4.6 Macrophages as Therapeutic Targets: Translational Frontiers

**FIGURE 4:**
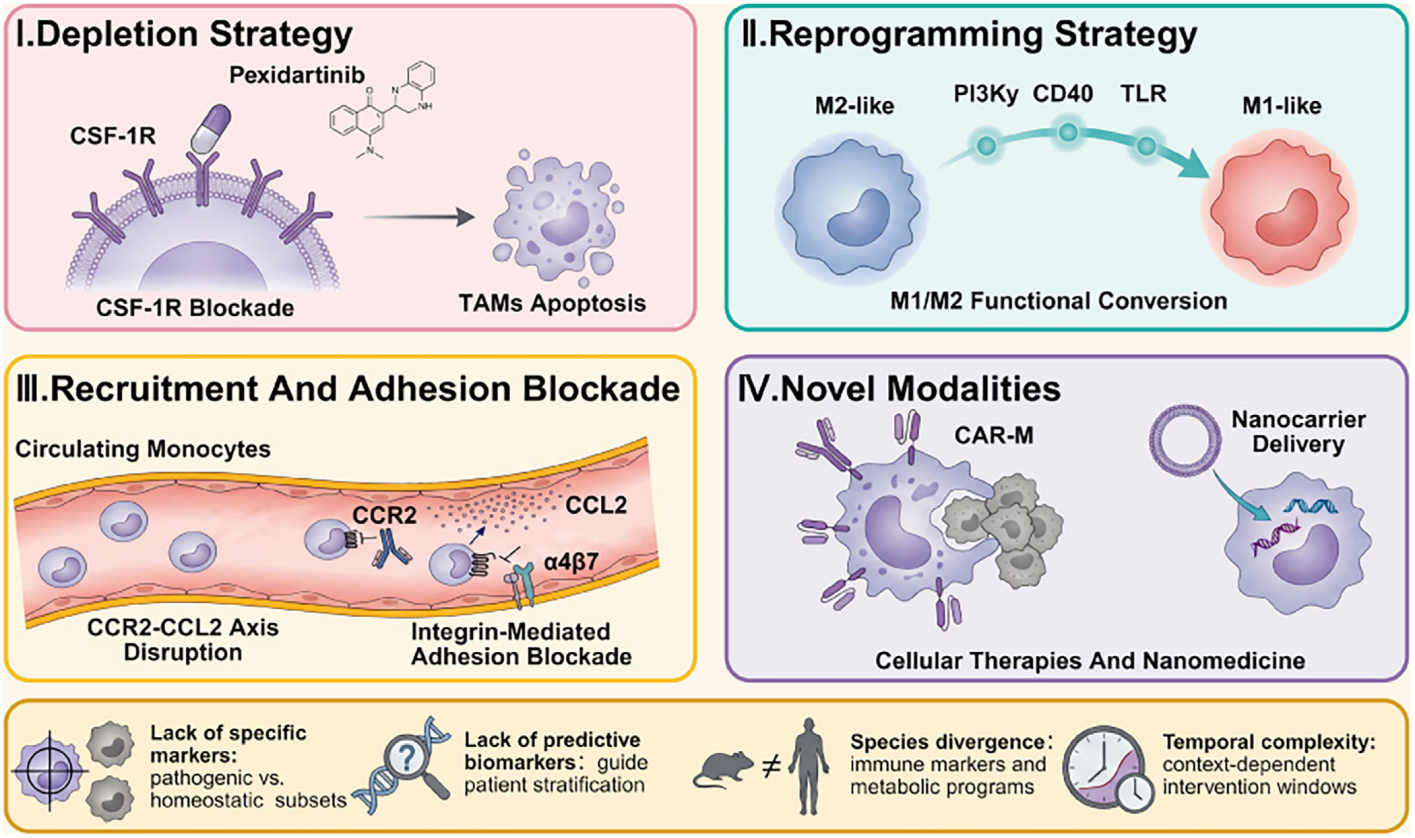
Therapeutic targeting of macrophages: clinical strategies and translational bottlenecks. (I) Depletion strategies eliminate pathogenic macrophages by blocking CSF‐1R signaling, as exemplified by the small‐molecule inhibitor Pexidartinib, which induces TAM apoptosis. (II) Reprogramming strategies restore immune homeostasis by converting M2‐like macrophages into an M1‐like antitumor phenotype, with key targets including PI3Kγ, CD40, and TLRs. (III) Recruitment and adhesion blockade interrupts the CCR2–CCL2 chemokine axis and integrin‐mediated adhesion (e.g., α4β7 blockade), thereby preventing circulating monocyte extravasation and infiltration into diseased tissues. (IV) Novel modalities encompass CAR‐M capable of targeted tumor phagocytosis and biomimetic nanocarrier systems enabling macrophage‐specific delivery of drugs or nucleic acids. Despite this expanding therapeutic arsenal, four critical translational bottlenecks persist: the absence of specific markers distinguishing pathogenic from homeostatic macrophage subsets; the lack of predictive biomarkers to guide patient stratification; significant species divergence in immune markers and metabolic programs between mice and humans; and the temporal complexity of context‐dependent intervention windows. (Created in https://BioRender.com).

Given their central driving role across a spectrum of diseases and their remarkable functional plasticity, macrophages have emerged as a prominent therapeutic target in translational medicine [215, 216]. Current macrophage‐directed strategies principally encompass the selective depletion of pathogenic subsets, reprogramming of functional phenotypes, and blockade of their recruitment to diseased tissues (Figure **4**). Recent breakthroughs in genetic engineering and nanodelivery technologies have further enabled the clinical evaluation of novel modalities, such as chimeric antigen receptor macrophages (CAR‐M) and biomimetic nanoparticles. Nevertheless, several persistent bottlenecks continue to impede clinical translation, including insufficient targeting specificity, a dearth of predictive biomarkers, the challenge of defining optimal intervention windows, and the translational gap arising from species divergence. This chapter systematically delineates the mechanistic principles and clinical progress of these strategies and explores future directions for precisely modulating macrophage function to restore immune homeostasis.

### 4.7 Depletion Strategies

The rationale underlying macrophage depletion strategies is that the sustained accumulation of pathogenic macrophages at disease sites serves as a central driver of disease progression, and that selectively eliminating these cells can directly interrupt inflammatory signaling and tissue damage. A core target of this strategy is the colony‐stimulating factor 1 receptor (CSF‐1R) pathway, which sustains macrophage tissue residence and functional activity by regulating their survival, proliferation, and differentiation. The small‐molecule CSF‐1R inhibitor pexidartinib has received FDA approval for the treatment of tenosynovial giant cell tumors, where it reduces pro‐tumor macrophages within the tumor microenvironment by blocking CSF‐1R signaling [217]. However, dose selection is a critical determinant of efficacy. Recent preclinical studies have demonstrated that high‐dose CSF‐1R inhibition can paradoxically induce the expansion of immunosuppressive macrophage populations and accelerate tumor growth, whereas low‐dose regimens may yield superior therapeutic outcomes. This observation partly explains the failure of such agents to improve patient survival as monotherapy in solid tumors such as pancreatic cancer. In posttransplant maintenance therapy for multiple myeloma, combining a CSF‐1R inhibitor with lenalidomide eliminates CD64^+^CD169^+^CD163^+^ immunosuppressive macrophages, enhances T cell activation markers, and delays disease progression [218]. Clodronate liposomes, widely used as a depletion tool in experimental studies, alleviate adipose tissue inflammation and improve insulin sensitivity in obese mice by inducing macrophage apoptosis; however, their clinical translation remains constrained by insufficient targeting precision and potential systemic toxicity.

### 4.8 Reprogramming Strategies

Macrophage reprogramming strategies aim to restore tissue immune homeostasis by targeting polarization‐associated signaling pathways to shift pro‐inflammatory or pro‐tumorigenic macrophages toward anti‐inflammatory or antitumor phenotypes [219, 220]. Unlike depletion or recruitment blockade approaches, reprogramming preserves the homeostatic functions of macrophages while correcting their aberrant activation, thereby reducing the risk of infection and immunosuppression. CD40, a key reprogramming target, is a receptor that, upon engagement by CD40L on activated T cells, upregulates co‐stimulatory molecules and pro‐inflammatory cytokines. Agonistic CD40 antibodies such as selicrelumab promote pro‐inflammatory differentiation of monocytes, enhance antigen presentation, and suppress tumor growth while prolonging survival in melanoma and colorectal cancer models. In local delivery systems for triple‐negative breast cancer, combining a CD40 agonist with chemotherapy and immune checkpoint inhibitors not only circumvents the systemic toxicities potentially associated with systemic administration but also enhances the activation of PD‐1^+^CD8^+^ T cells [221]. Furthermore, intratumoral injection of TLR agonists can reprogram macrophages from an immunosuppressive M2‐like phenotype to an antitumor M1‐like phenotype, showing promising potential in preclinical solid tumor models.

### 4.9 Recruitment and Adhesion Blockade

Blocking monocyte recruitment and adhesion to diseased tissues represents an upstream strategy for limiting the accumulation of pathogenic macrophages. Its rationale is that, within chronic inflammatory and tumor microenvironments, chemokine gradients generated by locally produced chemokines and their receptors continuously drive circulating monocytes to migrate toward pathological sites, where they subsequently differentiate into pathogenic macrophages. Core targets of this strategy include the CCL2/CCR2 chemokine axis and integrin‐mediated adhesion cascades. In recent clinical studies, Cenicriviroc, a dual CCR2/CCR5 antagonist, demonstrated significant antifibrotic efficacy in a phase IIb trial for nonalcoholic steatohepatitis [222]. Monocyte adhesion and transendothelial migration are mediated by integrins. In this context, the oral α4β7 antagonist Emvistegrast (GS‐1427) is currently being evaluated in a phase II trial for ulcerative colitis, where it is designed to exert anti‐inflammatory effects by blocking α4β7‐mediated lymphocyte homing to the gut [223]. Furthermore, the 7aaRGD peptide can prevent the formation of glioma‐associated immunosuppressive macrophages and promote vascular normalization by blocking SPP1‐integrin signaling, and its combination with anti‐PD‐1 antibodies restores CD8^+^ T cell proliferation and interferon‐γ production [224]. However, the clinical efficacy of this approach has fallen short of preclinical expectations. This is largely because CCL2/CCR2 blockade only suppresses further monocyte recruitment without eliminating macrophages that have already infiltrated and established residence in diseased tissues, which can continue to drive disease progression. Moreover, treatment discontinuation can trigger a rapid influx of circulating monocytes back into pathological sites, causing a swift rebound in macrophage numbers and accelerating disease deterioration.

### 4.10 Novel Modalities: Cellular Therapies and Nanomedicine

Novel macrophage‐targeted therapies are overcoming the limitations of conventional strategies through advances in genetic engineering and nanodelivery systems [225, 226, 227]. CAR‐M are engineered to specifically recognize and phagocytose tumor cells [228]. CD3ζ‐based CARs drive targeted phagocytosis and tumor killing in human macrophages via a Syk‐dependent mechanism without requiring additional opsonization factors. The chimeric adenoviral vector Ad5f35 can transduce primary human monocytes and macrophages with an efficiency exceeding 75%; the resulting CAR‐M cells exhibit potent tumor clearance capacity both in vitro and in vivo, migrating to primary tumors and pulmonary metastases after intravenous infusion and significantly prolonging overall survival in xenograft models. Importantly, Ad5f35 transduction confers a durable M1 antitumor phenotype on CAR‐M cells that resists conversion even upon exposure to M2‐polarizing signals, and it enables the reprogramming of M2 macrophages toward an M1 phenotype, underscoring their resilience against the immunosuppressive microenvironment [229]. In the treatment of glioblastoma, CAR‐M engineered with a sialic acid‐binding Ig‐like lectin 9 (SIGLEC9)‐based synthetic chimeric switch receptor converts inhibitory signals into TLR4 and CD40 activating signals, thereby sustaining a pro‐inflammatory phenotype and eliciting durable antitumor immune memory [230]. The phase I clinical trial of the HER2‐targeted CAR‐macrophage therapy CT‐0508 has provided preliminary validation of the feasibility and safety of this platform [231]. In metabolic diseases and chronic inflammation, nanomedicine delivery systems offer precision tools for macrophage‐targeted interventions. Dextran‐shell microparticles achieve macrophage‐specific delivery of siRNA or small‐molecule drugs through Dectin‐1 receptor‐mediated phagocytosis, effectively silencing inflammatory genes in adipose tissue macrophages and improving systemic insulin sensitivity in obese mouse models [232, 233]. Macrophage membrane‐coated biomimetic nanoparticles, by mimicking the immune evasion and lesion‐homing properties of native macrophages, demonstrate the dual advantages of precise delivery and immunomodulation in diseases such as atherosclerosis, inflammatory bowel disease, and acute pancreatitis. However, the clinical translation of these novel modalities must still surmount critical challenges, including scalable manufacturing, delivery efficiency, off‐target effects, and long‐term safety.

### 4.11 Clinical Translation Overview

Multiple macrophage‐targeted therapies have progressed from preclinical proof‐of‐concept to clinical validation across a range of disease indications (Table **2**). CSF‐1R blockade has already received regulatory approval for tenosynovial giant cell tumor, demonstrating that selective macrophage depletion can yield clear clinical benefit in specific indications. Reprogramming strategies, including CD40 agonists, PI3K‐γ inhibitors, and TREM2‐modulating antibodies, are currently in phase I/II clinical trials. Despite this progress, however, significant translational bottlenecks persist, which can be grouped into four key areas. First, most existing agents target pan‐macrophage markers and cannot distinguish protective from pathogenic subsets; indiscriminate targeting thus frequently disrupts tissue homeostasis and elevates the risk of infection. Second, there is a paucity of reliable biomarkers capable of reflecting macrophage polarization status and functional orientation in real time, making it difficult to identify which patients are suitable candidates for targeted therapy, to determine the optimal intervention window, or to dynamically monitor therapeutic efficacy. Third, macrophages display fundamentally distinct functional orientations at different disease stages; mild early‐stage inflammation may be protective, whereas blanket blockade throughout the disease course can interfere with the differentiation of pro‐resolving phenotypes. The precise timing of intervention and the choice of strategy must therefore be dynamically calibrated according to disease stage, yet current clinical protocols largely lack such temporal considerations. Fourth, substantial differences between murine and human macrophages in surface markers, functional properties, and metabolic programs mean that strategies effective in preclinical models frequently fail to translate into human trials. The lack of humanized models and ex vivo functional assay platforms further restricts both predictive biomarker development and drug screening efficiency. Overcoming these challenges will require single‐cell atlas‐guided identification of pathogenic subset‐specific markers to enable precision targeting, together with multi‐omics integration to establish predictive biomarker panels and achieve real‐time monitoring of macrophage polarization states for precise calibration of intervention timing. Moreover, constructing humanized models to bridge the species translational gap constitutes another critical step toward improving clinical translation efficiency. Thus, although macrophage‐targeted therapy holds considerable promise, its clinical translation continues to face substantial obstacles. Future investigations in more refined disease models are needed to drive the development of superior targeted agents, with the ultimate goal of restoring the dynamic equilibrium of macrophage activation states and thereby reestablishing tissue immune homeostasis.

**TABLE 2:** Representative clinical trials of macrophage‐targeting strategies.

Indication	Target	Agent	Mechanism	Clinical Trial ID	Current phase
TGCT	CSF‐1R	Pexidartinib (TURALIO)	Depletion	NCT02371369	FDA‐approved
Advanced solid tumors	CSF‐1R	Pexidartinib	Depletion	NCT02452424	Phase I
Obesity‐related insulin resistance	Macrophages	Clodronate Liposomes	Depletion	—	Preclinical
Resectable pancreatic cancer	CD40	Selicreluab (RG7876)	Reprogramming	NCT02588443	Phase I
BCL	CD40	Selicreluab	Reprogramming	NCT03892525	Phase I
Advanced solid tumors	PI3K‐γ	Eganelisib (IPI‐549)	Reprogramming	NCT02637531	Phase I/II
TNBC/RCC	PI3K‐γ	Eganelisib	Reprogramming	NCT03961698	Phase II
PROC/Advanced solid tumors	TREM2	PY314	Depletion	NCT04691375	Phase I
T2DM	GLP‐1R	Liraglutide	Reprogramming	—	Clinical proof concept
NASH	CCR2/CCR5	Cenicriviroc (CVC)	Recruitment/Adhesion	NCT02217475	Phase IIb
UC	Integrin α4β7	Emvistegrast (GS‐1427)	Recruitment/Adhesion	NCT06290934	Phase II
HER2^+^ solid tumors	HER2‐CAR	CT‐0508 (CAR‐M)	Cellular engineering	NCT04660929	Phase I

Abbreviations: BCL: B‐cell lymphoma; NASH: NASH with liver fibrosis; PROC: platinum‐resistant ovarian cancer; RCC: renal cell carcinoma; T2DM: type 2 diabetes; TGCT: tenosynovial giant cell tumor; TNBC: triple‐negative breast cancer; UC: ulcerative colitis. (Records obtained from clinical trial at http://clinicaltrials.gov).

## 5 Challenges and Future Perspectives

Although macrophage research has progressed from morphological description to molecular dissection at single‐cell resolution, multiple bottlenecks persist along the trajectory from fundamental understanding to clinical translation. These bottlenecks are deeply interconnected and collectively constitute a major impediment to advancing the field.

Developmental origin and heterogeneity resolution represent the foremost challenge. Single‐cell and spatial transcriptomics have identified over a dozen functionally distinct macrophage subsets within tissues, yet these technologies remain constrained by significant limitations. Cell recovery during tissue dissociation is suboptimal; cellular debris generated in the process can interfere with downstream analyses, and the dissociation procedure itself may inadvertently activate cells and alter their transcriptional states [234]. More problematically, different research groups employ their own nomenclature systems to define macrophage subsets, resulting in a lack of standardized terminology that impedes cross‐study data comparison and integration. Moreover, existing studies have predominantly focused on subset classification per se, with insufficient functional characterization at the subset level. This makes it difficult to determine whether a given subset represents an independent cell population or merely a transitional state within the classical activation spectrum. The limited conservation of markers across species further compounds this challenge; for example, CLEC4F, commonly used to define mouse Kupffer cells, is not expressed in humans.

The traditional M1/M2 dichotomy in polarization and plasticity research presents another fundamental obstacle. Although this framework has greatly facilitated our understanding of macrophage biology, M1‐ and M2‐associated markers can be co‐expressed by individual cells in vivo, and certain markers lack clear boundaries in distinguishing pro‐inflammatory from anti‐inflammatory phenotypes. Prolonged reliance on this simplified paradigm may obscure the true activation states and functional plasticity of macrophages. As high‐throughput stimulation experiments have revealed, macrophage activation more closely resembles a multidimensional landscape, with at least nine distinct activation trajectories directed toward divergent functional outcomes. How to move beyond the binary framework and construct a dynamic classification system that captures the continuous activation spectrum has therefore become a pressing theoretical bottleneck.

Furthermore, the study of macrophage function lacks spatiotemporal resolution. Although macrophage functional orientation is dynamically reshaped across disease stages, current investigations remain predominantly static and have failed to systematically capture longitudinal functional trajectories over the full disease course. Reliable biomarkers capable of reflecting macrophage polarization status and functional orientation in real time remain critically scarce, directly limiting our ability to pinpoint the pathogenic roles of macrophages at distinct disease stages. In parallel, while immunometabolism and developmental biology of macrophages have each made substantial progress over the past decade, how these two dimensions reciprocally shape one another remains poorly understood. Whether macrophages of distinct developmental origins rely on different metabolic programs to sustain their functional plasticity, and how metabolic reprogramming calibrates functional orientation across subsets of different origins, are questions of critical importance for designing precision‐targeted strategies, yet systematic investigations remain conspicuously sparse. At the clinical translation level, even when selective elimination of pathogenic subsets is achieved, whether the organism rapidly replenishes the depleted pool through accelerated monocyte differentiation or local proliferation is largely unexplored. More critically, whether macrophages can develop acquired resistance to long‐term therapy, analogous to tumor cells, has yet to enter the mainstream research agenda. Moreover, the clinical prospects of therapeutic strategies that merely seek to increase the proportion of a given polarization phenotype without achieving precision modulation of specific functional subsets are bound to be constrained.

To overcome these bottlenecks, future research must advance synergistically along several fronts. At the technical and conceptual level, establishing a unified macrophage nomenclature system, developing low‐damage tissue dissociation and functional preservation technologies, and constructing cross‐species comparable classification frameworks are urgent priorities. At the mechanistic level, integrating single‐cell multi‐omics with functional perturbation approaches is needed to move from descriptive subset cataloging toward causal dissection of subset functions. At the translational level, concerted efforts should be devoted to developing biomarker panels capable of real‐time, dynamic monitoring of macrophage functional states and to designing stage‐specific interventional protocols tailored to different phases of disease. Only by integrating developmental origins, polarization plasticity, metabolic regulation, and functional outputs within a unified spatiotemporal framework can we achieve precise calibration of macrophage function, thereby establishing a solid theoretical foundation for therapeutic strategies aimed at restoring immune homeostasis.

## 6 Conclusion

Macrophages are both central guardians of organismal homeostasis and one of the most intricate double‐edged swords in disease pathogenesis. Their functional identity is not rigidly predetermined by developmental origin but is instead dynamically shaped and continuously calibrated by the signaling networks within the tissue microenvironment. Thus, macrophages are not intrinsically “protective” or “pathogenic”; rather, their functional consequences depend entirely on the congruence between a given activation state and the prevailing pathological context. When microenvironmental signals become disrupted and the M1/M2 polarization balance breaks down, homeostatic guardians are transformed into disease drivers, and this functional skewing constitutes a common immunological foundation for diverse pathological processes, including cancer, autoimmune diseases, metabolic disorders, and fibrosis. Accordingly, macrophage‐targeted therapeutic strategies should not aim at indiscriminate elimination but should instead center on correcting functional skewing and restoring dynamic equilibrium. Current intervention strategies, represented by selective depletion, functional reprogramming, and blockade of monocyte recruitment, have demonstrated considerable potential for reestablishing immune homeostasis in both preclinical studies and early‐phase clinical trials. However, bottlenecks, including insufficient target selectivity, a dearth of reliable biomarkers, and species divergence between preclinical models and human biology, continue to profoundly limit the efficiency of clinical translation. As single‐cell multi‐omics technologies resolve macrophage heterogeneity at ever‐increasing depth, and as humanized models and dynamic monitoring platforms become increasingly sophisticated, the precise targeting of pathogenic subsets to calibrate activation states and restore immune equilibrium holds the promise of moving from concept to clinical practice.

## Author Contributions

Bihang Sun: Writing – original draft. Linqing Wen: Data source, Writing – original draft. Shiyun Tang: Data source. Lu Liu: Data source. Nianzhi Chen: Conceptualization, supervision. All authors have read and approved the final manuscript.

## Funding

The study received funding from the National Natural Science Foundation of China (Grant Nos. 82505686, 82474684), the China Postdoctoral Science Foundation (Grant No. 2023MD734135), and the Natural Science Foundation of Chongqing (Grant No. CSTB2023NSCQ‐BHX0019).

## Conflicts of Interest

The authors declare no conflicts of interest.

## Ethics Statement

The authors have nothing to report.

## Data Availability

The authors have nothing to report.

## References

[1] A. Shapouri‐Moghaddam , S. Mohammadian , H. Vazini , et al., “Macrophage Plasticity, Polarization, and Function in Health and Disease,” Journal of Cellular Physiology 233, no. 9 (2018): 6425–6440. 10.1002/jcp.26429 PMC13482560 29319160

[2] M. D. Cooper and M. N. Alder , “The Evolution of Adaptive Immune Systems,” Cell 124, no. 4 (2006): 815–822. 16497590 10.1016/j.cell.2006.02.001

[3] L. Bosurgi , A. A. Manfredi , and P. Rovere‐Querini , “Macrophages in Injured Skeletal Muscle: A Perpetuum Mobile Causing and Limiting Fibrosis, Prompting or Restricting Resolution and Regeneration,” Frontiers in immunology 2 (2011): 62. 22566851 10.3389/fimmu.2011.00062 PMC3341990

[4] R. Jin , J. Hao , Y. Yi , E. Sauter , and B. Li , “Regulation of Macrophage Functions by FABP‐Mediated Inflammatory and Metabolic Pathways,” Biochimica et Biophysica Acta (BBA)—Molecular and Cell Biology of Lipids 1866, no. 8 (2021): 158964. 33984518 10.1016/j.bbalip.2021.158964 PMC8169605

[5] S. Verheijden , S. De Schepper , and G. E. Boeckxstaens , “Neuron‐Macrophage Crosstalk in the Intestine: A “Microglia” Perspective,” Frontiers in Cellular Neuroscience 9 (2015): 403. 26528133 10.3389/fncel.2015.00403 PMC4603243

[6] P. Schädel , A. Czapka , N. Gebert , I. D. Jacobsen , A. Ori , and O. Werz , “Metabololipidomic and Proteomic Profiling Reveals Aberrant Macrophage Activation and Interrelated Immunomodulatory Mediator Release During Aging,” Aging Cell 22, no. 7 (2023): e13856. 37101405 10.1111/acel.13856 PMC10352559

[7] Y. Wang , X. Lu , J. Lu , P. Hernigou , and F. Jin , “The Role of Macrophage Polarization in Tendon Healing and Therapeutic Strategies: Insights From Animal Models,” Frontiers in Bioengineering and Biotechnology 12 (2024): 1366398. 38486869 10.3389/fbioe.2024.1366398 PMC10937537

[8] H. Lu , J. Huang , W. Y. chao , E. Casals , G. Casals , and M. Zeng , “Beyond M1/M2: The Role of Reactive Oxygen Species in Liver Fibrosis and Immune Modulation,” Redox Biology 88 (2025): 103933. 41260098 10.1016/j.redox.2025.103933 PMC12670537

[9] V. M. T. Bart , R. J. Pickering , P. R. Taylor , and N. Ipseiz , “Macrophage Reprogramming for Therapy,” Immunology 163, no. 2 (2021): 128–144. 33368269 10.1111/imm.13300 PMC8114216

[10] L. Hui , Y. Li , M. K. Huang , Y. M. Jiang , and T. Liu , “CXCL13: A Common Target for Immune‐Mediated Inflammatory Diseases,” Clinical and Experimental Medicine 24, no. 1 (2024): 244. 39443356 10.1007/s10238-024-01508-8 PMC11499446

[11] X. Li , W. Gao , X. Long , and M. Wu , “New Insights Into Monocyte‐Derived Macrophages in Glioblastoma,” Research 8 (2025): 0836. 40800581 10.34133/research.0836 PMC12340225

[12] W. Wang , Z. Yi , Z. Yang , et al., “The Hepatic Macrophage: A Key Regulator of Liver Metastatic Tumor Microenvironment Through Cell Crosstalk,” Journal of Translational Medicine 23, no. 1 (2025): 1334. 41272833 10.1186/s12967-025-07376-4 PMC12639949

[13] S. Wang , S. Wang , H. Chen , and J. Xu , “Microglia–Neuron Crosstalk: An Intimate Molecular Conversation in Neurodegeneration,” International Journal of Molecular Sciences 27, no. 4 (2026): 2011. 41752147 10.3390/ijms27042011 PMC12940662

[14] M. D. Park , A. Silvin , F. Ginhoux , and M. Merad , “Macrophages in Health and Disease,” Cell 185, no. 23 (2022): 4259–4279. 36368305 10.1016/j.cell.2022.10.007 PMC9908006

[15] Y. Lavin and M. Merad , “Macrophages: Gatekeepers of Tissue Integrity,” Cancer Immunology Research 1, no. 4 (2013): 201–209. 24777851 10.1158/2326-6066.CIR-13-0117 PMC4144820

[16] F. Ginhoux and M. Guilliams , “Tissue‐Resident Macrophage Ontogeny and Homeostasis,” Immunity 44, no. 3 (2016): 439–449. 26982352 10.1016/j.immuni.2016.02.024

[17] Q. Wang and W. Ma , “Revisiting TAM Polarization: Beyond M1‐ and M2‐type TAM Toward Clinical Precision in Macrophage‐targeted Therapy,” Experimental and Molecular Pathology 143 (2025): 104982. 40664070 10.1016/j.yexmp.2025.104982

[18] B. Malissen , S. Tamoutounour , and S. Henri , “The Origins and Functions of Dendritic Cells and Macrophages in the Skin,” Nature Reviews Immunology 14, no. 6 (2014): 417–428. 10.1038/nri3683 24854591

[19] T. Lazarov , S. Juarez‐Carreño , N. Cox , and F. Geissmann , “Physiology and Diseases of Tissue‐Resident Macrophages,” Nature 618, no. 7966 (2023): 698–707. 37344646 10.1038/s41586-023-06002-x PMC10649266

[20] M. N. Artyomov , A. Sergushichev , and J. D. Schilling , “Integrating Immunometabolism and Macrophage Diversity,” Seminars in Immunology 28, no. 5 (2016): 417–424. 27771140 10.1016/j.smim.2016.10.004 PMC5333784

[21] X. Huang , X. Yang , L. Xiang , and Y. Chen , “Serine Metabolism in Macrophage Polarization,” Inflammation Research Springer Science and Business Media Deutschland GmbH 73, no. 1 (2024): 83–98. 10.1007/s00011-023-01815-y 38070057

[22] N. Luque‐Campos , F. A. Bustamante‐Barrientos , C. Pradenas , et al., “The Macrophage Response Is Driven by Mesenchymal Stem Cell‐Mediated Metabolic Reprogramming,” Frontiers in Immunology 12 (2021): 624746. 34149687 10.3389/fimmu.2021.624746 PMC8213396

[23] T. Zhao , Y. Wang , and F. A. Lin , “Identification of Key Macrophage‐Related Genes in Systemic Sclerosis–Associated Interstitial Lung Disease Based on Single‐Cell and Bulk Transcriptomic Data,” PLoS ONE 21, no. 3 (2026): e0344166. 41785251 10.1371/journal.pone.0344166 PMC12962529

[24] S. Härtle , K. Sutton , L. Vervelde , and T. S. Dalgaard , “Delineation of Chicken Immune Markers in the Era of Omics and Multicolor Flow Cytometry,” Frontiers in Veterinary Science 11 (2024): 1385400. 38846783 10.3389/fvets.2024.1385400 PMC11156169

[25] A. A. Filardy , J. R. M. Ferreira , R. M. Rezende , B. L. Kelsall , and R. P. Oliveira , “The Intestinal Microenvironment Shapes Macrophage and Dendritic Cell Identity and Function,” Immunology Letters 253 (2023): 41–53. 36623708 10.1016/j.imlet.2023.01.003 PMC9907447

[26] D. Y. Guo , Z. Y. Liu , X. C. Xu , et al., “Neutrophil Heterogeneity in Airway Inflammatory Diseases,” Inflammation 48, no. 6 (2025): 3800–3827. 40826205 10.1007/s10753-025-02351-z PMC12722436

[27] T. Yin , X. Li , Y. Li , X. Zang , L. Liu , and M. Du , “Macrophage Plasticity and Function in Cancer and Pregnancy,” Frontiers in immunology 14 (2024): 1333549. 38274812 10.3389/fimmu.2023.1333549 PMC10808357

[28] A. Hassanshahi , M. Moradzad , S. Ghalamkari , M. Fadaei , A. J. Cowin , and M. Hassanshahi , “Macrophage‐Mediated Inflammation in Skin Wound Healing,” Cells 11, no. 19 (2022): 2953. 36230913 10.3390/cells11192953 PMC9564023

[29] Z. Woolf , M. E. V. Swanson , and L. C. Smyth , “Single‐cell Image Analysis Reveals a Protective Role for Microglia in Glioblastoma,” Neuro‐Oncology Advances 3, no. 1 (2021): vdab031. 34286275 10.1093/noajnl/vdab031 PMC8284623

[30] J. Hou , Y. Chen , G. Grajales‐Reyes , and M. Colonna , “TREM2 dependent and Independent Functions of Microglia in Alzheimer's Disease,” Molecular Neurodegeneration 17, no. 1 (2022): 84. 36564824 10.1186/s13024-022-00588-y PMC9783481

[31] K. Man , V. I. Kutyavin , and A. Chawla , “Tissue Immunometabolism: Development, Physiology, and Pathobiology,” Cell Metabolism 25, no. 1 (2017): 11–26. 27693378 10.1016/j.cmet.2016.08.016 PMC5226870

[32] G. Hoeffel and F. Ginhoux , “Fetal Monocytes and the Origins of Tissue‐Resident Macrophages,” Cellular Immunology 330 (2018): 5–15. 29475558 10.1016/j.cellimm.2018.01.001

[33] R. Watanabe and M. Hashimoto , “Pathogenic Role of Monocytes/Macrophages in Large Vessel Vasculitis,” Frontiers in Immunology 13 (2022): 859502. 35967455 10.3389/fimmu.2022.859502 PMC9372263

[34] F. O. Martinez , T. W. Combes , F. Orsenigo , and S. Gordon , “Monocyte Activation in Systemic Covid‐19 Infection: Assay and Rationale,” EBioMedicine 59 (2020): 102964. 32861199 10.1016/j.ebiom.2020.102964 PMC7456455

[35] N. Xu , B. A. Gonzalez , and K. E. Yutzey , “Macrophage Lineages in Heart Development and Regeneration,” Current Topics in Developmental Biology 156 (2024): 1–17. 38556420 10.1016/bs.ctdb.2024.01.004 PMC13551468

[36] B. Kanuri , K. P. Maremanda , D. Chattopadhyay , et al., “Redefining Macrophage Heterogeneity in Atherosclerosis: A Focus on Possible Therapeutic Implications,” Comprehensive Physiology 15, no. 2 (2025): e70008. 40108774 10.1002/cph4.70008

[37] H. Ma , M. Zhu , M. Chen , X. Li , and X. Feng , “The Role of Macrophage Plasticity in Neurodegenerative Diseases,” Biomarker Research 12, no. 1 (2024): 81. 39135084 10.1186/s40364-024-00624-7 PMC11321226

[38] C. C. Bain and A. S. MacDonald , “The Impact of the Lung Environment on Macrophage Development, Activation and Function: Diversity in the Face of Adversity,” Mucosal Immunol 15, no. 2 (2022): 223–234. 35017701 10.1038/s41385-021-00480-w PMC8749355

[39] K. S. Park , Y. J. Ko , and J. H. Choi , “Hypoxia‐inducible Factors Link Inflammation and Lipid Metabolism in Atherosclerotic Macrophages,” Frontiers in Cardiovascular Medicine 13 (2026): 1765661. 41736827 10.3389/fcvm.2026.1765661 PMC12926842

[40] Y. Chen , Q. Zhu , A. Yin , W. Wang , and J. Wang , “Triptolide‐mediated Immunomodulation of Macrophages: From Pathophysiology to Therapy,” Annals of Medicine 57, no. 1 (2025): 2575302. 41144250 10.1080/07853890.2025.2575302 PMC12570235

[41] P. Kolypetri and H. L. Weiner , “Monocyte Regulation by Gut Microbial Signals,” Trends in Microbiology 31, no. 10 (2023): 1044–1057. 37271658 10.1016/j.tim.2023.05.006 PMC10524398

[42] J. M. Ortiz Wilczyñski , H. A. Mena , M. M. Ledesma , et al., “The Synthetic Phospholipid C8‐C1P Determines Pro‐Angiogenic and Pro‐Reparative Features in human Macrophages Restraining the Proinflammatory M1‐Like Phenotype,” Frontiers in Immunology 14 (2023): 1162671. 37398671 10.3389/fimmu.2023.1162671 PMC10311553

[43] R. Gentek , K. Molawi , and M. H. Sieweke , “Tissue Macrophage Identity and Self‐Renewal,” Immunological Reviews 262, no. 1 (2014): 56–73. 25319327 10.1111/imr.12224

[44] Y. Sun , J. Li , X. Xie , et al., “Macrophage‐Osteoclast Associations: Origin, Polarization, and Subgroups,” Frontiers in Immunology 12 (2021): 778078. 34925351 10.3389/fimmu.2021.778078 PMC8672114

[45] X. Gao , Z. Cao , H. Tan , et al., “LncRNA, an Emerging Approach for Neurological Diseases Treatment by Regulating Microglia Polarization,” Frontiers in Neuroscience 16 (2022): 903472. 35860297 10.3389/fnins.2022.903472 PMC9289270

[46] D. Chen , J. Hu , M. Zhu , et al., “C‐reactive Protein Is a Broad‐spectrum Capsule‐binding Receptor for Hepatic Capture of Blood‐borne Bacteria,” Embo Journal 44, no. 24 (2025): 7364–7394. 41214213 10.1038/s44318-025-00623-w PMC12705745

[47] L. Rabiller , V. Robert , A. Arlat , et al., “Driving Regeneration, Instead of Healing, in Adult Mammals: The Decisive Role of Resident Macrophages Through Efferocytosis,” NPJ Regenerative Medicine 6, no. 1 (2021): 41. 34344890 10.1038/s41536-021-00151-1 PMC8333253

[48] J. Cao , Y. Zhang , S. Guo , et al., “Immune Biomarkers in Circulating Cells of NSCLC Patients Can Effectively Evaluate the Efficacy of Chemotherapy Combined With Anti‐PD‐1 Therapy,” Frontiers in Immunology 16 (2025): 1521708. 40308600 10.3389/fimmu.2025.1521708 PMC12040615

[49] M. Shojaei , B. Frey , F. Putz , R. Fietkau , U. S. Gaipl , and A. Derer , “Chemoradiation‐Altered Micromilieu of Glioblastoma Cells Particularly Impacts M1‐Like Macrophage Activation,” International Journal of Molecular Sciences 26, no. 14 (2025): 6574. 40724825 10.3390/ijms26146574 PMC12294741

[50] O. M. A. Dagah , B. B. Silaa , M. Zhu , et al., “Exploring Immune Redox Modulation in Bacterial Infections: Insights Into Thioredoxin‐Mediated Interactions and Implications for Understanding Host–Pathogen Dynamics,” Antioxidants 13, no. 5 (2024): 545. 38790650 10.3390/antiox13050545 PMC11117976

[51] Y. Zheng , Y. Wang , J. Li , et al., “PGAM5 Modulates Macrophage Polarization, Aggravating Inflammation in COPD via the NF‐κB Pathway,” International, Peer‐Reviewed Journal of Therapeutics and Pharmacology 20 (2025): 551–564. 10.2147/COPD.S492627 PMC11897911 40078929

[52] M. Hulsmans and M. Nahrendorf , “Smad3 Cranks up the Appetite of Infarct Macrophages,” Circulation Research 125, no. 1 (2019): 71–73. 31219739 10.1161/CIRCRESAHA.119.315306 PMC6588180

[53] C. Zhao , T. X. Medeiros , R. J. Sové , B. H. Annex , and A. S. Popel , “A Data‐Driven Computational Model Enables Integrative and Mechanistic Characterization of Dynamic Macrophage Polarization,” Iscience 24, no. 2 (2021): 102112. 33659877 10.1016/j.isci.2021.102112 PMC7895754

[54] M. Zhao , S. Yu , M. Zhang , et al., “Macrophages in Ulcerative Colitis: Immunomodulatory Roles, Phenotypic Switching, and Therapeutic Targeting,” Journal of Innate Immunity 18, no. 1 (2026): 85–103. 41528944 10.1159/000550397 PMC12923256

[55] X. Xu , D. K. W. Ocansey , B. Pei , et al., “Resveratrol Alleviates DSS‐Induced IBD in Mice by Regulating the Intestinal Microbiota‐Macrophage‐Arginine Metabolism Axis,” European Journal of Medical Research 28, no. 1 (2023): 319. 37660064 10.1186/s40001-023-01257-6 PMC10474707

[56] J. Le , Y. Kulatheepan , and S. Jeyaseelan , “Role of Toll‐Like Receptors and Nod‐Like Receptors in Acute Lung Infection,” Frontiers in Immunology 14 (2023): 1249098. 37662905 10.3389/fimmu.2023.1249098 PMC10469605

[57] F. Jyotsna , J. Ikram , F. Nageeta , et al., “Unlocking the Potential of Immunotherapy in Cardiovascular Disease: A Comprehensive Review of Applications and Future Directions,” Cureus 15, no. 8 (2023): e42790, Published online August 1, 2023. 37664375 10.7759/cureus.42790 PMC10469982

[58] Z. Liu and W. Xu , “Neutrophil and Macrophage Response in Acinetobacter Baumannii Infection and Their Relationship to Lung Injury,” Frontiers in Cellular and Infection Microbiology 12 (2022): 890511. 35873147 10.3389/fcimb.2022.890511 PMC9298752

[59] Y. Xu , K. Hu , C. Liu , et al., “Eschar Dissolution and the Immunoregulator Effect of Keratinase on Burn Wounds,” Scientific Reports 13, no. 1 (2023): 13238. 37580372 10.1038/s41598-023-39765-4 PMC10425458

[60] P. J. Murray , “Macrophage Polarization,” Annual Review of Physiology 79 (2017): 541–566. 10.1146/annurev-physiol-022516-034339 27813830

[61] S. Yu , S. Wang , X. Wang , and X. Xu , “The Axis of Tumor‐associated Macrophages, Extracellular Matrix Proteins, and Cancer‐associated Fibroblasts in Oncogenesis,” Cancer cell international 24, no. 1 (2024): 335. 39375726 10.1186/s12935-024-03518-8 PMC11459962

[62] G. Ma , Z. Zhang , P. Li , et al., “Reprogramming of Glutamine Metabolism and Its Impact on Immune Response in the Tumor Microenvironment,” Cell Communication and Signaling 20, no. 1 (2022): 114. 35897036 10.1186/s12964-022-00909-0 PMC9327201

[63] M. K. Callaway , B. J. Noonan , K. L. Schwertfeger , and P. P. Provenzano , “Extracellular Matrix Architecture Promotes Immunosuppressive Microenvironments in Pancreatic Cancer,” Matrix Biology 141 (2025): 114–126. 40975454 10.1016/j.matbio.2025.09.004 PMC12536408

[64] M. van Eijk and J. Aerts , “The Unique Phenotype of Lipid‐Laden Macrophages,” International Journal of Molecular Sciences 22, no. 8 (2021): 4039. 33919858 10.3390/ijms22084039 PMC8070766

[65] H. R. Chang , T. Josefs , D. Scerbo , et al., “Role of LpL (Lipoprotein Lipase) in Macrophage Polarization in Vitro and in Vivo,” Arteriosclerosis, Thrombosis, and Vascular Biology 39, no. 10 (2019): 1967–1985. 31434492 10.1161/ATVBAHA.119.312389 PMC6761022

[66] B. S. Finlin , B. Zhu , C. P. Starnes , R. E. McGehee , C. A. Peterson , and P. A. Kern , “Regulation of Thrombospondin‐1 Expression in Alternatively Activated Macrophages and Adipocytes: Role of Cellular Cross Talk and Omega‐3 Fatty Acids,” Journal of Nutritional Biochemistry 24, no. 9 (2013): 1571–1579. 23528972 10.1016/j.jnutbio.2013.01.007 PMC3695002

[67] H. Duan , L. Jing , J. Xiang , et al., “CD146 Associates With Gp130 to Control a Macrophage Pro‐Inflammatory Program That Regulates the Metabolic Response to Obesity,” Advanced Science 9, no. 13 (2022): e2103719. 35258174 10.1002/advs.202103719 PMC9069186

[68] A. Viola , F. Munari , R. Sánchez‐Rodríguez , T. Scolaro , and A. Castegna , “The Metabolic Signature of Macrophage Responses,” Frontiers in Immunology 10, no. JULY (2019): 1462. 31333642 10.3389/fimmu.2019.01462 PMC6618143

[69] R. Y. Ma , A. Black , and B. Z. Qian , “Macrophage Diversity in Cancer Revisited in the Era of Single‐Cell Omics,” Trends in Immunology 43, no. 7 (2022): 546–563. 35690521 10.1016/j.it.2022.04.008

[70] S. K. Wculek , G. Dunphy , I. Heras‐Murillo , A. Mastrangelo , and D. Sancho , “Metabolism of Tissue Macrophages in Homeostasis and Pathology,” Cellular & Molecular Immunology 19, no. 3 (2022): 384–408. 34876704 10.1038/s41423-021-00791-9 PMC8891297

[71] M. Yao , M. Li , D. Peng , et al., “Unraveling Macrophage Polarization: Functions, Mechanisms, and “Double‐Edged Sword” Roles in Host Antiviral Immune Responses,” International Journal of Molecular Sciences 25, no. 22 (2024): 12078. 39596148 10.3390/ijms252212078 PMC11593441

[72] S. Z. Berg and J. Berg , “Microbes, Macrophages, and Melanin: A Unifying Theory of Disease as Exemplified by Cancer,” Frontiers in Immunology 15 (2025): 1493978. 39981299 10.3389/fimmu.2024.1493978 PMC11840190

[73] Y. Wang , N. Ding , L. Qi , W. Chen , and P. Wu , “Immunopharmacology of Gastric Cancer–Deciphering Immune Cell Subset Responses and Nanoparticle‐Mediated Targeting,” Frontiers in Pharmacology 16 (2025): 1611234. 40458806 10.3389/fphar.2025.1611234 PMC12127324

[74] D. A. Hume , S. M. Millard , and A. R. Pettit , “Macrophage Heterogeneity in the Single‐Cell Era: Facts and Artifacts,” Blood 142, no. 16 (2023): 1339–1347. 37595274 10.1182/blood.2023020597

[75] L. Dou , X. Shi , X. He , and Y. Gao , “Macrophage Phenotype and Function in Liver Disorder,” Frontiers in Immunology 10 (2020): 3112. 32047496 10.3389/fimmu.2019.03112 PMC6997484

[76] Z. Fremont‐Debaene and S. Faure‐Dupuy , “Macrophage Makeover Extreme Viral Edition: Mechanisms of Immune Subversion and Therapeutic Perspectives,” Journal of General Virology 107, no. 2 (2026): 002228. 41729693 10.1099/jgv.0.002228 PMC12928704

[77] M. F. Viola and G. Boeckxstaens , “Niche‐specific Functional Heterogeneity of Intestinal Resident Macrophages,” Gut 70, no. 7 (2021): 1383–1395. 33384336 10.1136/gutjnl-2020-323121 PMC8223647

[78] Q. Zhang , Q. Song , Z. Li , X. Wu , Y. Chen , and H. Lin , “Targeting Fibroblasts in Pathological Bone Formation: Mechanisms and Treatments,” Frontiers in Cell and Developmental Biology 13 (2025): 1612950. 40491950 10.3389/fcell.2025.1612950 PMC12146285

[79] I. Rosa , E. Romano , B. S. Fioretto , and M. Manetti , “Pathophysiologic Implications and Therapeutic Potentials of Telocytes in Multiorgan Fibrosis,” Current Opinion in Rheumatology 38, no. 1 (2026): 26–37. 40747598 10.1097/BOR.0000000000001116 PMC12672042

[80] L. Xu , C. Huang , X. Zheng , et al., “Elevated CD169 Expressing Monocyte/Macrophage Promotes Systemic Inflammation and Disease Progression in Cirrhosis,” Clinical and Experimental Medicine 24, no. 1 (2024): 45. 38413535 10.1007/s10238-024-01305-3 PMC10899294

[81] S. Schuermans , C. Kestens , and P. E. Marques , “Systemic Mechanisms of Necrotic Cell Debris Clearance,” Cell Death & Disease 15, no. 8 (2024): 557. 39090111 10.1038/s41419-024-06947-5 PMC11294570

[82] H. Ishikura , H. Okada , Y. Kin , et al., “Loss of Mechanical Stress Induces Synovitis, Fibrosis and Articular Cartilage Degeneration via Distinct Synovial Cell Subsets,” Scientific Reports 16, no. 1 (2026): 8007. 41663755 10.1038/s41598-026-39416-4 PMC12957370

[83] P. Xia , Y. Qu , Q. Liu , et al., “Identification of Programmed Cell Death‐Related Subtypes Reveals Immune Heterogeneity and Therapeutic Divergence in Colon Cancer,” Theranostics 16, no. 9 (2026): 4821–4840. 41799193 10.7150/thno.126314 PMC12964230

[84] A. K. Elfstrum , A. S. Bapat , and K. L. Schwertfeger , “Defining and Targeting Macrophage Heterogeneity in the Mammary Gland and Breast Cancer,” Cancer Medicine 13, no. 3 (2024): e7053. 38426622 10.1002/cam4.7053 PMC10905685

[85] D. Jiang , R. Xiao , J. Bai , et al., “FOLR2+ macrophages in Cancer: Allies or Enemies,” Cell Communication and Signaling 23, no. 1 (2025): 261. 40457348 10.1186/s12964-025-02257-1 PMC12131471

[86] X. Liao , E. Chang , X. Tang , et al., “Cardiac Macrophages Regulate Isoproterenol‐Induced Takotsubo‐Like Cardiomyopathy,” JCI Insight 7, no. 3 (2022): e156236. 35132957 10.1172/jci.insight.156236 PMC8855841

[87] R. Chen , Y. Xie , Z. Chang , et al., “Integration of Single‐Cell Sequencing With Machine Learning and Mendelian Randomization Analysis Identifies the NAP1L1 Gene as a Predictive Biomarker for Alzheimer's Disease,” Front Aging Neurosci 16, (2024): 1406160. 38988327 10.3389/fnagi.2024.1406160 PMC11233722

[88] M. Locati , G. Curtale , and A. Mantovani , “Diversity, Mechanisms, and Significance of Macrophage Plasticity,” Annual Review of Pathology 15 (2020): 123–147, Published online 2019. 10.1146/annurev-pathmechdis-012418-012718 PMC7176483 31530089

[89] X. Fu , M. Pang , Z. Wang , and H. Wang , “Macrophage Polarization in the Tumor Microenvironment of Hepatocellular Carcinoma: From Mechanistic Insights to Translational Therapies,” Cancer Control 32 (2025): 10732748251406674. 41403016 10.1177/10732748251406674 PMC12709032

[90] Y. Zhang , X. Yang , T. Wang , et al., “Transcriptomic Profiling Reveals Tissue‐Specific and Sex‐Dimorphic Lipid Storage in *Bufo gargarizans* ,” BMC Genomics [Electronic Resource] 26, no. 1 (2025): 1028. 41219836 10.1186/s12864-025-12221-5 PMC12606946

[91] A. Hu , L. Sun , H. Lin , Y. Liao , H. Yang , and Y. Mao , “Harnessing Innate Immune Pathways for Therapeutic Advancement in Cancer,” Signal Transduction and Targeted Therapy 9, no. 1 (2024): 68. 38523155 10.1038/s41392-024-01765-9 PMC10961329

[92] J. Van den Bossche , L. A. O'Neill , and D. Menon , “Macrophage Immunometabolism: Where Are We (Going)?,” Trends in Immunology 38, no. 6 (2017): 395–406. 28396078 10.1016/j.it.2017.03.001

[93] S. Yao , Y. Cen , G. Lou , and Y. Liu , “Therapeutic Potentials of Mesenchymal Stem Cells and Their Extracellular Vesicles on Liver Diseases by Modulating Mitochondrial Function of Macrophages,” International Immunopharmacology 165 (2025): 115486. 40925200 10.1016/j.intimp.2025.115486

[94] X. Che , Y. Zhang , X. Chen , et al., “The Lactylation‐Macrophage Interplay: Implications for Gastrointestinal Disease Therapeutics,” Frontiers in Immunology 16 (2025): 1608115. 40703527 10.3389/fimmu.2025.1608115 PMC12283307

[95] J. Saravia , J. L. Raynor , N. M. Chapman , S. A. Lim , and H. Chi , “Signaling Networks in Immunometabolism,” Cell Research 30, no. 4 (2020): 328–342. 32203134 10.1038/s41422-020-0301-1 PMC7118125

[96] A. C. Boese and S. Kang , “Mitochondrial Metabolism‐Mediated Redox Regulation in Cancer Progression,” Redox Biology 42 (2021): 101870. 33509708 10.1016/j.redox.2021.101870 PMC8113029

[97] Y. Zhang , Z. Liu , and H. Sun , “Fetal‐Maternal Interactions During Pregnancy: A ‘Three‐in‐One’ Perspective,” Frontiers in Immunology 14 (2023): 1198430. 37350956 10.3389/fimmu.2023.1198430 PMC10282753

[98] S. Yu , J. Fu , J. Wang , et al., “The Influence of Mitochondrial‐DNA‐Driven Inflammation Pathways on Macrophage Polarization: A New Perspective for Targeted Immunometabolic Therapy in Cerebral Ischemia‐Reperfusion Injury,” International Journal of Molecular Sciences 23, no. 1 (2021): 135. 35008558 10.3390/ijms23010135 PMC8745401

[99] L. Wang , D. Wang , T. Zhang , Y. Ma , X. Tong , and H. Fan , “The Role of Immunometabolism in Macrophage Polarization and Its Impact on Acute Lung Injury/Acute Respiratory Distress Syndrome,” Frontiers in Immunology 14 (2023): 1117548. 37020557 10.3389/fimmu.2023.1117548 PMC10067752

[100] L. Sainero‐Alcolado , J. Liaño‐Pons , M. V. Ruiz‐Pérez , and M. Arsenian‐Henriksson , “Targeting Mitochondrial Metabolism for Precision Medicine in Cancer,” Cell Death and Differentiation 29, no. 7 (2022): 1304–1317. 35831624 10.1038/s41418-022-01022-y PMC9287557

[101] Y. Wang , X. Zhang , S. Wang , et al., “Identification of Metabolism‐Associated Biomarkers for Early and Precise Diagnosis of Oral Squamous Cell Carcinoma,” Biomolecules 12, no. 3 (2022): 400. 35327590 10.3390/biom12030400 PMC8945702

[102] D. S. Koenis , L. Medzikovic , P. B. van Loenen , et al., “Nuclear Receptor Nur77 Limits the Macrophage Inflammatory Response Through Transcriptional Reprogramming of Mitochondrial Metabolism,” Cell Reports 24, no. 8 (2018): 2127–2140.e7. 30134173 10.1016/j.celrep.2018.07.065 PMC6113932

[103] A. Qadeer , A. Ullah , M. Z. Khan , et al., “Extracellular Vesicles Associated Metabolites as Intercellular Signalling Mediators in Disease and Therapy,” Metabolites 16, no. 3 (2026): 207. 41893356 10.3390/metabo16030207 PMC13028176

[104] S. N. Bess , M. J. Igoe , A. C. Denison , and T. J. Muldoon , “Autofluorescence Imaging of Endogenous Metabolic Cofactors in Response to Cytokine Stimulation of Classically Activated Macrophages,” Cancer & Metabolism 11, no. 1 (2023): 22. 37957679 10.1186/s40170-023-00325-z PMC10644562

[105] C. Di , X. Chu , P. Chang , et al., “The Roles of Histone H3K18 Lactylation, Acetylation, and Lactylation/Acetylation Ratio as Potential Biomarkers in the Diagnosis and Severity Assessment of Sepsis and Septic Shock,” Infectious Diseases and Therapy 14, no. 12 (2025): 2785–2818. 41085943 10.1007/s40121-025-01232-0 PMC12602787

[106] Y. Zhou , Y. Yuan , X. Yao , et al., “miPEP31 Alleviates Sepsis Development by Regulating Chi3l1‐Dependent Macrophage Polarization,” Biology Direct 19, no. 1 (2024): 117. 39558383 10.1186/s13062-024-00568-w PMC11575066

[107] J. Cheng , Y. Zhang , L. Ma , et al., “Macrophage‐Derived Extracellular Vesicles‐Coated Palladium Nanoformulations Modulate Inflammatory and Immune Homeostasis for Targeting Therapy of Ulcerative Colitis,” Advanced Science 10, no. 33 (2023): e2304002. 37807805 10.1002/advs.202304002 PMC10667822

[108] J. Yan and T. Horng , “Lipid Metabolism in Regulation of Macrophage Functions,” Trends in Cell Biology 30, no. 12 (2020): 979–989. 33036870 10.1016/j.tcb.2020.09.006

[109] A. J. Mouton , X. Li , M. E. Hall , and J. E. Hall , “Obesity, Hypertension, and Cardiac Dysfunction Novel Roles of Immunometabolism in Macrophage Activation and Inflammation,” Circulation Research 126, no. 6 (2020): 789–806. 32163341 10.1161/CIRCRESAHA.119.312321 PMC7255054

[110] F. Vinchi , “Macrophage‐Based Cell Strategies: A Novel Approach in Immunotherapy,” Hemasphere 6, no. 2 (2022): e682. 35198857 10.1097/HS9.0000000000000682 PMC8855737

[111] X. Chen , C. Lai , L. Cai , and L. Huang , “Cross One Single Body 49 Tissues Single‐Cell Transcriptome Reveals Detailed Macrophage Heterogeneity During Pig Pregnancy,” Frontiers in Immunology 16 (2025): 1574120. 40242774 10.3389/fimmu.2025.1574120 PMC12000058

[112] J. Bonnardel and M. Guilliams , “Developmental Control of Macrophage Function,” Current Opinion in Immunology 50 (2018): 64–74. 29247852 10.1016/j.coi.2017.12.001

[113] E. Boada‐Romero , J. Martinez , B. L. Heckmann , and D. R. Green , “The Clearance of Dead Cells by Efferocytosis,” Nature Reviews Molecular Cell Biology 21, no. 7 (2020): 398–414. 32251387 10.1038/s41580-020-0232-1 PMC7392086

[114] C. Chen , J. Wang , C. Liu , and J. Hu , “Cardiac Resident Macrophages: Key Regulatory Mediators in the Aftermath of Myocardial Infarction,” Frontiers in Immunology 14 (2023): 1207100. 37457720 10.3389/fimmu.2023.1207100 PMC10348646

[115] R. Pandit and A. Yurdagul , “The Atherosclerotic Plaque Microenvironment as a Therapeutic Target,” Current Atherosclerosis Reports 27, no. 1 (2025): 47. 40172727 10.1007/s11883-025-01294-y PMC11965263

[116] P. Sun , S. Liu , Q. Zeng , et al., “The Regulatory Mechanisms of *Treponema pallidum* Enolase on Macrophages: From Enzymatic Activity to Signal Transduction,” The FASEB Journal 39, no. 13 (2025): e70801. 40600943 10.1096/fj.202500358R PMC12219466

[117] H. Li , B. Sun , X. Ning , S. Jiang , and L. Sun , “A Comparative Analysis of Edwardsiella Tarda‐Induced Transcriptome Profiles in RAW264.7 Cells Reveals New Insights Into the Strategy of Bacterial Immune Evasion,” International Journal of Molecular Sciences 20, no. 22 (2019): 5724. 31731575 10.3390/ijms20225724 PMC6888325

[118] J. Correale and E. Carnero Contentti , “Induction of Immune Tolerance in NMOSD and MOGAD,” Therapeutic Advances in Neurological Disorders 18 (2025): 17562864251357393. 40761287 10.1177/17562864251357393 PMC12319201

[119] H. Shah , Z. Liu , W. Guo , W. Ren , and Y. Xiao , “Immune‐Regulating Extracellular Vesicles: A New Frontier in Autoimmune Disease Therapy,” Essays in Biochemistry 69, no. 02 (2025): 161–168. 40366303 10.1042/EBC20253016 PMC12224888

[120] R. Huang , T. Kang , and S. Chen , “The Role of Tumor‐Associated Macrophages in Tumor Immune Evasion,” Journal of Cancer Research and Clinical Oncology 150, no. 5 (2024): 238. 38713256 10.1007/s00432-024-05777-4 PMC11076352

[121] K. M. Sheu and A. Hoffmann , “Functional Hallmarks of Healthy Macrophage Responses: Their Regulatory Basis and Disease Relevance,” Annual Review of Immunology 40 (2022): 295–321. 10.1146/annurev-immunol-101320-031555 PMC10074967 35471841

[122] M. K. Khoury , H. Yang , and B. Liu , “Macrophage Biology in Cardiovascular Diseases,” Arteriosclerosis, Thrombosis, and Vascular Biology 41, no. 2 (2021): E77–E81. 10.1161/ATVBAHA.120.313584 PMC8046835 33054391

[123] F. Geissmann and E. Mass , “A Stratified Myeloid System, the Challenge of Understanding Macrophage Diversity,” Seminars in Immunology 27, no. 6 (2015): 353–356. 27038773 10.1016/j.smim.2016.03.016 PMC4968038

[124] E. R. Stevenson , L. C. Smith , M. L. Wilkinson , S. J. Lee , and A. J. Gow , “Etiology of Lipid‐Laden Macrophages in the Lung,” International Immunopharmacology 123 (2023): 110719. 37595492 10.1016/j.intimp.2023.110719 PMC10734282

[125] J. Kang , P. Hua , X. Wu , and B. Wang , “Exosomes: Efficient Macrophage‐Related Immunomodulators in Chronic Lung Diseases,” Frontiers in Cell and Developmental Biology 12 (2024): 1271684. 38655063 10.3389/fcell.2024.1271684 PMC11035777

[126] G. I. Soma , M. Oda , V. T. Tjhin , C. Kohchi , and H. Inagawa , “Oral and Transdermal Administration of Lipopolysaccharide Safely Enhances Self‐Healing Ability Through the Macrophage Network,” Frontiers in Immunology 16 (2025): 1563484. 40230835 10.3389/fimmu.2025.1563484 PMC11994614

[127] P. R. Taylor , L. Martinez‐Pomares , M. Stacey , H. H. Lin , G. D. Brown , and S. Gordon , “Macrophage Receptors and Immune Recognition,” Annual Review of Immunology 23 (2005): 901–944. 10.1146/annurev.immunol.23.021704.115816 15771589

[128] S. Yadav , A. Priya , D. R. Borade , and R. Agrawal‐Rajput , “Macrophage Subsets and Their Role: Co‐Relation With Colony‐Stimulating Factor‐1 Receptor and Clinical Relevance,” Immunologic Research 71, no. 2 (2023): 130–152. 36266603 10.1007/s12026-022-09330-8 PMC9589538

[129] Y. Q. Liu , Z. Z. Li , Y. L. Han , and Q. B. Wang , “The Role of Efferocytosis in Inflammatory Bowel Disease,” Frontiers in Immunology 16 (2025): 1524058. 40040696 10.3389/fimmu.2025.1524058 PMC11876057

[130] H. Pan , J. Liu , Y. Dong , et al., “Release of Prostaglandin E _1_ From *N* ‐(2‐Hydroxypropyl)methacrylamide Copolymer Conjugates by Bone Cells,” Macromolecular Bioscience 8, no. 7 (2008): 599–605. 18401866 10.1002/mabi.200700338 PMC4605216

[131] S. De Schepper , S. Verheijden , J. Aguilera‐Lizarraga , et al., “Self‐Maintaining Gut Macrophages Are Essential for Intestinal Homeostasis,” Cell 175, no. 2 (2018): 400–415.e13. 30173915 10.1016/j.cell.2018.07.048

[132] D. Lanjewar , R. Katariya , V. Kale , B. Taksande , M. Umekar , and M. Vinchurney , “Interplay of Neuropeptide Y and Autophagy in Alzheimer's Disease: Therapeutic Perspectives and Mechanistic Insights,” Neuropeptides 113 (2025): 102547. 40695083 10.1016/j.npep.2025.102547

[133] A. F. M. Salvador , N. Abduljawad , and J. Kipnis , “Meningeal Lymphatics in Central Nervous System Diseases,” Annual Review of Neuroscience 47, no. 1 (2024): 323–344. 10.1146/annurev-neuro-113023-103045 PMC12051392 38648267

[134] T. Zhao , Q. Jiang , W. Li , et al., “Antigen‐Presenting Cell‐Like Neutrophils Foster T Cell Response in Hyperlipidemic Patients and Atherosclerotic Mice,” Frontiers in Immunology 13 (2022): 851713. 35251050 10.3389/fimmu.2022.851713 PMC8891125

[135] S. N. Karagiannis , D. H. Josephs , H. J. Bax , and J. F. Spicer , “Therapeutic IgE Antibodies: Harnessing a Macrophage‐Mediated Immune Surveillance Mechanism Against Cancer,” Cancer Research 77, no. 11 (2017): 2779–2783. 28526770 10.1158/0008-5472.CAN-17-0428

[136] T. T. He , P. Y. Tang , X. L. Jiang , S. S. Sun , P. Nie , and H. X. Xie , “T3SS effector EseJ in *Edwardsiella piscicida* Inhibits PANoptosis in Macrophages,” Communications Biology 8, no. 1 (2025): 1420. 41044169 10.1038/s42003-025-08823-0 PMC12494976

[137] S. K. Tiwary , T. Hayashi , A. Kovacs , and D. L. Mann , “Recurrent Myocardial Injury Leads to Disease Tolerance in a Murine Model of Stress‐Induced Cardiomyopathy,” JACC: Basic to Translational Science 8, no. 7 (2023): 783–797. 37547073 10.1016/j.jacbts.2022.12.007 PMC10401155

[138] S. Dyckhoff‐Shen , I. Masouris , H. W. Pfister , et al., “Pharmacologic Depletion of Border‐Associated Macrophages Worsens Disease in a Mouse Model of Meningitis,” Acta Neuropathologica Communications 13, no. 1 (2025): 191. 40988032 10.1186/s40478-025-02126-5 PMC12455799

[139] A. Chavda , X. Zhou , M. A. Tokmedash , J. J. Moon , and J. Min , “Tunable Wrinkled Topographies Direct Dendritic Cell Maturation and Immune Phenotypes,” Acta Biomaterialia 212 (2026): 428–443. 41478520 10.1016/j.actbio.2025.12.050 PMC12997553

[140] N. Luo , G. Yang , P. Zhang , et al., “Development of an Early Mortality Risk Prediction Model for Pediatric Patients With Secondary Hemophagocytic Lymphohistiocytosis,” Italian Journal of Pediatrics 51, no. 1 (2025): 221. 40660317 10.1186/s13052-025-02084-7 PMC12257782

[141] P. Wielgat , K. Narejko , and H. Car , “SARS‐CoV‐2 Attacks in the Brain: Focus on the Sialome,” Cells 11, no. 9 (2022): 1458. 35563764 10.3390/cells11091458 PMC9104523

[142] I. M. De la Fuente , J. Carrasco‐Pujante , M. Fedetz , et al., “Migratory Responses in Enucleated Cells: The Forces Driving the Locomotion Movement of Unicellular Organisms,” PNAS Nexus 4, no. 8 (2025): pgaf232. 40799349 10.1093/pnasnexus/pgaf232 PMC12341899

[143] F. Constanty , B. Wu , K. H. Wei , et al., “Border‐zone Cardiomyocytes and Macrophages Regulate Extracellular Matrix Remodeling to Promote Cardiomyocyte Protrusion During Cardiac Regeneration,” Nature Communications 16, no. 1 (2025): 3823. 10.1038/s41467-025-59169-4 PMC12019606 40268967

[144] D. Moreno‐Blas , T. Adell , and C. González‐Estévez , “Autophagy in Tissue Repair and Regeneration,” Cells 14, no. 4 (2025): 282. 39996754 10.3390/cells14040282 PMC11853389

[145] H. Li , Q. Zhu , W. Wang , et al., “Identification of Biomarkers Associated With M1 Macrophages in the ST‐segment Elevation Myocardial Infarction Through Bioinformatics and Machine Learning Approaches,” Scientific Reports 15, no. 1 (2025): 11069. 40169697 10.1038/s41598-025-89125-7 PMC11961635

[146] T. D. Smith , R. R. Nagalla , E. Y. Chen , and W. F. Liu , “Harnessing Macrophage Plasticity for Tissue Regeneration,” Advanced Drug Delivery Reviews 114 (2017): 193–205. 28449872 10.1016/j.addr.2017.04.012

[147] T. A. Wynn and K. M. Vannella , “Macrophages in Tissue Repair, Regeneration, and Fibrosis,” Immunity 44, no. 3 (2016): 450–462. 26982353 10.1016/j.immuni.2016.02.015 PMC4794754

[148] P. Huebener and R. F. Schwabe , “Regulation of Wound Healing and Organ Fibrosis by Toll‐Like Receptors,” Biochimica et Biophysica Acta (BBA)—Molecular Basis of Disease 1832, no. 7 (2013): 1005–1017. 23220258 10.1016/j.bbadis.2012.11.017 PMC3848326

[149] S. Watanabe , M. Alexander , A. V. Misharin , and G. R. S. Budinger , “The Role of Macrophages in the Resolution of Inflammation,” Journal of Clinical Investigation 129, no. 7 (2019): 2619–2628. 31107246 10.1172/JCI124615 PMC6597225

[150] Y. Zheng , Y. Han , Q. Sun , and Z. Li , “Harnessing Anti‐Tumor and Tumor‐Tropism Functions of Macrophages via Nanotechnology for Tumor Immunotherapy,” Exploration 2, no. 3 (2022): 20210166. 37323705 10.1002/EXP.20210166 PMC10190945

[151] Z. Liu , Y. Li , Y. Ren , et al., “Efferocytosis: The Janus‐Faced Gatekeeper of Aging and Tumor Fate,” Aging Cell 24, no. 2 (2025): e14467. 39748782 10.1111/acel.14467 PMC11822654

[152] J. Luo , Q. Lian , D. Zhu , et al., “PLSCR1 Promotes Apoptosis and Clearance of Retinal Ganglion Cells in Glaucoma Pathogenesis,” Genes & Diseases 10, no. 4 (2023): 1564–1581. 37397520 10.1016/j.gendis.2022.05.036 PMC10311034

[153] N. Karaji and Q. J. Sattentau , “Efferocytosis of Pathogen‐Infected Cells,” Frontiers in Immunology 8 (2017): 1863. 29312342 10.3389/fimmu.2017.01863 PMC5743670

[154] B. L. Heckmann , E. Boada‐Romero , L. D. Cunha , J. Magne , and D. R. Green , “LC3‐Associated Phagocytosis and Inflammation,” Journal of Molecular Biology 429, no. 23 (2017): 3561–3576. 28847720 10.1016/j.jmb.2017.08.012 PMC5743439

[155] B. Krenz , J. Lee , T. Kannan , and M. Eilers , “Immune Evasion: An Imperative and Consequence of MYC Deregulation,” Molecular Oncology 18, no. 10 (2024): 2338–2355, Published online July 2, 2024. 38957016 10.1002/1878-0261.13695 PMC11459038

[156] H. Li , A. Wang , Y. Zhang , and F. Wei , “Diverse Roles of Lung Macrophages in the Immune Response to Influenza A Virus,” Frontiers in Microbiology 14 (2023): 1260543. 37779697 10.3389/fmicb.2023.1260543 PMC10534047

[157] R. Siebeler , M. P. J. de Winther , and M. A. Hoeksema , “The Regulatory Landscape of Macrophage Interferon Signaling in Inflammation,” Journal of Allergy and Clinical Immunology 152, no. 2 (2023): 326–337. 37271317 10.1016/j.jaci.2023.04.022

[158] M. Shu , M. Fang , T. Xu , and Q. Yan , “Functional Roles and Regulatory Mechanisms of Paeonol in the Treatment of Liver Disease,” Natural Products and Bioprospecting 16, no. 1 (2026): 2. 41491143 10.1007/s13659-025-00554-3 PMC12770108

[159] Y. Wang , X. Y. Liu , Y. Wang , et al., “NOX2 inhibition Stabilizes Vulnerable Plaques by Enhancing Macrophage Efferocytosis via MertK/PI3K/AKT Pathway,” Redox Biology 64 (2023): 102763. 37354827 10.1016/j.redox.2023.102763 PMC10320254

[160] D. Sokolova , T. Childs , and S. Hong , “Insight Into the Role of Phosphatidylserine in Complement‐Mediated Synapse Loss in Alzheimer's Disease,” Faculty Reviews 10 (2021): 19. 33718936 10.12703/r/10-19 PMC7946395

[161] C. Gupta , J. Xu , T. Jin , et al., “Single‐Cell Network Biology Characterizes Cell Type Gene Regulation for Drug Repurposing and Phenotype Prediction in Alzheimer's Disease,” Plos Computational Biology 18, no. 7 (2022): e1010287. 35849618 10.1371/journal.pcbi.1010287 PMC9333448

[162] R. A. Campbell , M. H. Docherty , D. A. Ferenbach , and K. J. Mylonas , “The Role of Ageing and Parenchymal Senescence on Macrophage Function and Fibrosis,” Frontiers in Immunology 12 (2021): 700790. 34220864 10.3389/fimmu.2021.700790 PMC8248495

[163] G. Urbani , E. Rondini , E. Distrutti , S. Marchianò , M. Biagioli , and S. Fiorucci , “Phenotyping the Chemical Communications of the Intestinal Microbiota and the Host: Secondary Bile Acids as Postbiotics,” Cells 14, no. 8 (2025): 595. 40277921 10.3390/cells14080595 PMC12025480

[164] J. Ghebrehiwet‐Kuflom , A. Mehta , P. Lim , S. Dahle , and R. R. Isseroff , “GLP‐1 Receptor Agonists as Emerging Modulators of Inflammation and Angiogenesis in Chronic Cutaneous Wound Healing,” Journal of Investigative Dermatology 145, no. 12 (2025): 2981–2988. 41081666 10.1016/j.jid.2025.08.045

[165] E. A. Kim , N. Kang , J. Kim , H. W. Yang , G. Ahn , and S. J. Heo , “Anti‐Inflammatory Effect of Turbo Cornutus Viscera Ethanolic Extract Against Lipopolysaccharide‐Stimulated Inflammatory Response via the Regulation of the JNK/NF‐kB Signaling Pathway in Murine Macrophage RAW 264.7 Cells and a Zebrafish Model: A Preliminary Study,” Foods 11, no. 3 (2022): 364. 35159514 10.3390/foods11030364 PMC8834147

[166] N. Suttawong , N. Witayavanitkul , M. Chayanupatkul , et al., “ *Gardenia jasminoides* Fruit Extract Ameliorates Non‐Alcoholic Steatohepatitis With Fibrosis by Modulating Inflammatory and Fibrogenic Pathways,” PLoS ONE 20, no. 10 (2025): e0333800. 41042784 10.1371/journal.pone.0333800 PMC12494252

[167] C. Maruyama , M. Uchiyama , A. Umezawa , et al., “A Cross‐Sectional Pilot Study on Association of Ready‐to‐Eat and Processed Food Intakes With Metabolic Factors, Serum Trans Fat and Phospholipid Fatty Acid Compositions in Healthy Japanese Adults,” Nutrients 16, no. 7 (2024): 1032. 38613065 10.3390/nu16071032 PMC11013905

[168] B. Burja , D. Paul , A. Tastanova , et al., “An Optimized Tissue Dissociation Protocol for Single‐Cell RNA Sequencing Analysis of Fresh and Cultured Human Skin Biopsies,” Frontiers in Cell and Developmental Biology 10 (2022): 872688. 35573685 10.3389/fcell.2022.872688 PMC9096112

[169] X. Zhang , L. Ji , and M. O. Li , “Control of Tumor‐associated Macrophage Responses by Nutrient Acquisition and Metabolism,” Immunity 56, no. 1 (2023): 14–31. 36630912 10.1016/j.immuni.2022.12.003 PMC9839308

[170] Q. Zhou , C. Xue , J. Man , et al., “Correlation of Tumor‐Associated Macrophage Infiltration in Glioblastoma With Magnetic Resonance Imaging Characteristics: A Retrospective Cross‐Sectional Study,” Quantitative Imaging in Medicine and Surgery 13, no. 9 (2023): 5958–5973. 37711787 10.21037/qims-23-126 PMC10498259

[171] K. Wu , K. Lin , X. Li , et al., “Redefining Tumor‐Associated Macrophage Subpopulations and Functions in the Tumor Microenvironment,” Frontiers in Immunology 11 (2020): 1731. 32849616 10.3389/fimmu.2020.01731 PMC7417513

[172] Y. Pan , Y. Yu , X. Wang , and T. Zhang , “Tumor‐Associated Macrophages in Tumor Immunity,” Frontiers in Immunology 11 (2020): 583084. 33365025 10.3389/fimmu.2020.583084 PMC7751482

[173] Q. Zhang , Y. Wei , Y. Li , and X. Jiao , “Low MARCO Expression Is Associated With Poor Survival in Patients With Hepatocellular Carcinoma Following Liver Transplantation,” Cancer Management and Research 14 (2022): 1935–1944. 35720642 10.2147/CMAR.S363219 PMC9200231

[174] Y. Shao and B. Lu , “The Crosstalk Between Circular RNAs and the Tumor Microenvironment in Cancer Metastasis,” Cancer Cell International 20, no. 1 (2020): 448. 32943996 10.1186/s12935-020-01532-0 PMC7488731

[175] X. Bai , Y. R. Guo , Z. M. Zhao , et al., “Macrophage Polarization in Cancer and Beyond: From Inflammatory Signaling Pathways to Potential Therapeutic Strategies,” Cancer Letters 625 (2025): 217772. 40324582 10.1016/j.canlet.2025.217772

[176] H. Wang , X. Wang , X. Zhang , and W. Xu , “The Promising Role of Tumor‐Associated Macrophages in the Treatment of Cancer,” Drug Resistance Updates 73 (2024): 101041. 38198845 10.1016/j.drup.2023.101041

[177] N. R. Anderson , N. G. Minutolo , S. Gill , and M. Klichinsky , “Macrophage‐Based Approaches for Cancer Immunotherapy,” Cancer Research 81, no. 5 (2021): 1201–1208. 33203697 10.1158/0008-5472.CAN-20-2990

[178] W. Hou , B. Yang , and H. Zhu , “Nanoparticle‐Based Therapeutic Strategies for Enhanced Pancreatic Ductal Adenocarcinoma Immunotherapy,” Pharmaceutics 14, no. 10 (2022): 2033. 36297467 10.3390/pharmaceutics14102033 PMC9607590

[179] T. Xia , M. Zhang , W. Lei , et al., “Advances in the Role of STAT3 in Macrophage Polarization,” Frontiers in Immunology 14 (2023): 1160719. 37081874 10.3389/fimmu.2023.1160719 PMC10110879

[180] K. Pomeyie , F. Abrokwah , D. Boison , et al., “Macrophage Immunometabolism Dysregulation and Inflammatory Disorders,” Biomedicine and Pharmacotherapy 188 (2025): 118142. 40378771 10.1016/j.biopha.2025.118142

[181] S. Wu , S. Zhao , L. Hai , et al., “Macrophage Polarization Regulates the Pathogenesis and Progression of Autoimmune Diseases,” Autoimmunity Reviews 24, no. 7 (2025): 103820. 40268127 10.1016/j.autrev.2025.103820

[182] M. Kurowska‐Stolarska and S. Alivernini , “Synovial Tissue Macrophages in Joint Homeostasis, Rheumatoid Arthritis and Disease Remission,” Nature Reviews Rheumatology 18, no. 7 (2022): 384–397. 35672464 10.1038/s41584-022-00790-8

[183] K. Kulakova , T. R. Lawal , E. Mccarthy , and A. Floudas , “The Contribution of Macrophage Plasticity to Inflammatory Arthritis and Their Potential as Therapeutic Targets,” Cells 13, no. 18 (2024): 1586. 39329767 10.3390/cells13181586 PMC11430612

[184] E. Keewan and S. A. Naser , “The Role of Notch Signaling in Macrophages During Inflammation and Infection: Implication in Rheumatoid Arthritis?,” Cells 9, no. 1 (2020): 111. 31906482 10.3390/cells9010111 PMC7016800

[185] S. Semenistaja , S. Skuja , A. Kadisa , and V. Groma , “Healthy and Osteoarthritis‐Affected Joints Facing the Cellular Crosstalk,” International Journal of Molecular Sciences 24, no. 4 (2023): 4120. 36835530 10.3390/ijms24044120 PMC9964755

[186] K. Zhang , J. Guo , W. Yan , and L. Xu , “Macrophage Polarization in Inflammatory Bowel Disease,” Cell Communication and Signaling 21, no. 1 (2023): 367. 38129886 10.1186/s12964-023-01386-9 PMC10734116

[187] Y. Zheng , Y. Yu , X. F. Chen , S. L. Yang , X. L. Tang , and Z. G. Xiang , “Intestinal Macrophage Autophagy and Its Pharmacological Application in Inflammatory Bowel Disease,” Frontiers in Pharmacology 12 (2021): 803686. 34899362 10.3389/fphar.2021.803686 PMC8652230

[188] E. J. Wang , M. Y. Wu , Z. Y. Ren , et al., “Targeting Macrophage Autophagy for Inflammation Resolution and Tissue Repair in Inflammatory Bowel Disease,” Burns Trauma 11 (2023): tkad004. 37152076 10.1093/burnst/tkad004 PMC10157272

[189] Y. Zheng , K. Wei , P. Jiang , et al., “Macrophage Polarization in Rheumatoid Arthritis: Signaling Pathways, Metabolic Reprogramming, and Crosstalk With Synovial Fibroblasts,” Frontiers in Immunology 15 (2024): 1394108. 38799455 10.3389/fimmu.2024.1394108 PMC11116671

[190] X. Fang , J. Yang , L. Yang , et al., “Multi‐omics Analysis Identified Macrophages as Key Contributors to Sex‐related Differences in Ulcerative Colitis,” Frontiers in Immunology 16 (2025): 1569271. 40636118 10.3389/fimmu.2025.1569271 PMC12238219

[191] Y. Cui , J. Chen , Z. Zhang , H. Shi , W. Sun , and Q. Yi , “The Role of AMPK in Macrophage Metabolism, Function and Polarisation,” Journal of Translational Medicine 21, no. 1 (2023): 892. 38066566 10.1186/s12967-023-04772-6 PMC10709986

[192] M. Appari , K. M. Channon , and E. McNeill , “Metabolic Regulation of Adipose Tissue Macrophage Function in Obesity and Diabetes,” Antioxid Redox Signaling 29, no. 3 (2018): 297–312. 10.1089/ars.2017.7060 PMC6012981 28661198

[193] D. Thomas and C. Apovian , “Macrophage Functions in Lean and Obese Adipose Tissue,” Metabolism 72 (2017): 120–143. 28641779 10.1016/j.metabol.2017.04.005 PMC5516622

[194] L. Ménégaut , A. Jalil , C. Thomas , and D. Masson , “Macrophage Fatty Acid Metabolism and Atherosclerosis: The Rise of PUFAs,” Atherosclerosis 291 (2019): 52–61. 31693943 10.1016/j.atherosclerosis.2019.10.002

[195] T. Chavakis , V. I. Alexaki , and A. W. Ferrante , “Macrophage Function in Adipose Tissue Homeostasis and Metabolic Inflammation,” Nature Immunology 24, no. 5 (2023): 757–766. 37012544 10.1038/s41590-023-01479-0

[196] M. X. Wu , I. V. Ustyugova , L. Han , and O. E. Akilov , “Immediate Early Response Gene X‐1, a Potential Prognostic Biomarker in Cancers,” Expert Opinion on Therapeutic Targets 17, no. 5 (2013): 593–606. 23379921 10.1517/14728222.2013.768234 PMC4381960

[197] D. Liddle , A. Hutchinson , H. Wellings , K. Power , L. Robinson , and J. Monk , “Integrated Immunomodulatory Mechanisms Through Which Long‐Chain n‐3 Polyunsaturated Fatty Acids Attenuate Obese Adipose Tissue Dysfunction,” Nutrients 9, no. 12 (2017): 1289. 29186929 10.3390/nu9121289 PMC5748740

[198] A. D. Ruggiero , C. C. C. Key , and K. Kavanagh , “Adipose Tissue Macrophage Polarization in Healthy and Unhealthy Obesity,” Frontiers in Nutrition 8 (2021): 625331. 33681276 10.3389/fnut.2021.625331 PMC7925825

[199] K. Drareni , J. F. Gautier , N. Venteclef , and F. Alzaid , “Transcriptional Control of Macrophage Polarisation in Type 2 Diabetes,” Seminars in Immunopathology 41, no. 4 (2019): 515–529. 31049647 10.1007/s00281-019-00748-1

[200] M. Ahmed , M. P. J. de Winther , and J. Van den Bossche , “Epigenetic Mechanisms of Macrophage Activation in Type 2 Diabetes,” Immunobiology 222, no. 10 (2017): 937–943. 27613200 10.1016/j.imbio.2016.08.011

[201] J. Fang , Y. Wu , H. Wang , J. Zhang , and L. You , “Oral Health and Diabetic Cardiomyopathy: Mechanisms, Biomarkers, and Early Screening Approaches,” Journal of Inflammation Research 18 (2025): 8689–8704. 40626037 10.2147/JIR.S521430 PMC12230323

[202] W. Sheng , G. Ji , and L. Zhang , “Role of Macrophage Scavenger Receptor MSR1 in the Progression of Non‐Alcoholic Steatohepatitis,” Frontiers in Immunology 13 (2022): 1050984. 36591228 10.3389/fimmu.2022.1050984 PMC9797536

[203] A. Adnan , M. Juntunen , and T. Tyrväinen , “Effects of Bariatric Surgery‐Related Weight Loss on the Characteristics, Metabolism, and Immunomodulation of Adipose Stromal/Stem Cells in a Follow‐Up Study,” Stem Cells International 2025, no. 1 (2025): 1212255. 40395977 10.1155/sci/1212255 PMC12092157

[204] J. Q. Ban , L. H. Ao , X. He , H. Zhao , and J. Li , “Advances in Macrophage‐Myofibroblast Transformation in Fibrotic Diseases,” Frontiers in Immunology 15 (2024): 1461919. 39445007 10.3389/fimmu.2024.1461919 PMC11496091

[205] B. W. Zhou , H. M. Liu , F. Xu , and X. H. Jia , “The Role of Macrophage Polarization and Cellular Crosstalk in the Pulmonary Fibrotic Microenvironment: A Review,” Cell Communication and Signaling 22, no. 1 (2024): 172. 38461312 10.1186/s12964-024-01557-2 PMC10924385

[206] L. Bertran , A. Eigbefoh‐Addeh , M. Portillo‐Carrasquer , et al., “Identification of the Potential Molecular Mechanisms Linking RUNX1 Activity With Nonalcoholic Fatty Liver Disease, by Means of Systems Biology,” Biomedicines 10, no. 6 (2022): 1315. 35740337 10.3390/biomedicines10061315 PMC9219880

[207] W. L. Xun , Z. S. Xi , W. H. Juan , R. X. Lu , and J. Guo , “M2b macrophage Polarization and Its Roles in Diseases,” Journal of Leukocyte Biology 106, no. 2 (2019): 345–358. 30576000 10.1002/JLB.3RU1018-378RR PMC7379745

[208] C. Wang , C. Ma , L. Gong , et al., “Macrophage Polarization and Its Role in Liver Disease,” Frontiers in Immunology 12 (2021): 803037. 34970275 10.3389/fimmu.2021.803037 PMC8712501

[209] M. M. Aychman , D. L. Goldman , and J. S. Kaplan , “Cannabidiol's Neuroprotective Properties and Potential Treatment of Traumatic Brain Injuries,” Frontiers in Neurology 14 (2023): 1087011. 36816569 10.3389/fneur.2023.1087011 PMC9932048

[210] S. Yu , D. Kong , B. Lu , et al., “Mesenchymal Stem Cell‐Derived Exosomes as Cell‐Free Therapeutics: Mechanistic Insights and Engineering Strategies for Liver Disease Treatment,” Stem Cell Research & Therapy 16, no. 1 (2025): 652. 41261428 10.1186/s13287-025-04747-y PMC12632147

[211] D. Zhou , K. Yang , L. Chen , et al., “Macrophage Polarization and Function: New Prospects for Fibrotic Disease,” Immunology and Cell Biology 95, no. 10 (2017): 864–869. 29044201 10.1038/icb.2017.64

[212] C. Atri , F. Z. Guerfali , and D. Laouini , “Role of human Macrophage Polarization in Inflammation During Infectious Diseases,” International Journal of Molecular Sciences 19, no. 6 (2018): 1801. 29921749 10.3390/ijms19061801 PMC6032107

[213] G. A. Duque and A. Descoteaux , “Macrophage Cytokines: Involvement in Immunity and Infectious Diseases,” Frontiers in Immunology 5, no. OCT (2014): 491. 25339958 10.3389/fimmu.2014.00491 PMC4188125

[214] V. Torraca , S. Masud , H. P. Spaink , and A. H. Meijer , “Macrophage‐Pathogen Interactions in Infectious Diseases: New Therapeutic Insights From the Zebrafish Host Model,” DMM Disease Models and Mechanisms 7, no. 7 (2014): 785–797. 24973749 10.1242/dmm.015594 PMC4073269

[215] R. Chen , H. Zhang , B. Tang , et al., “Macrophages in Cardiovascular Diseases: Molecular Mechanisms and Therapeutic Targets,” Signal Transduction and Targeted Therapy 9, no. 1 (2024): 130. 38816371 10.1038/s41392-024-01840-1 PMC11139930

[216] S. Chen , A. Saeed , Q. Liu , et al., “Macrophages in Immunoregulation and Therapeutics,” Signal Transduction and Targeted Therapy 8, no. 1 (2023): 207. 37211559 10.1038/s41392-023-01452-1 PMC10200802

[217] M. Holterhus , B. Altvater , S. Kailayangiri , and C. Rossig , “The Cellular Tumor Immune Microenvironment of Childhood Solid Cancers: Informing More Effective Immunotherapies,” Cancers (Basel) 14, no. 9 (2022): 2177. 35565307 10.3390/cancers14092177 PMC9105669

[218] A. L. Garfall , A. D. Cohen , S. P. Susanibar‐Adaniya , et al., “Anti‐BCMA/CD19 CAR T Cells With Early Immunomodulatory Maintenance for Multiple Myeloma Responding to Initial or Later‐Line Therapy,” Blood Cancer Discovery 4, no. 2 (2023): 118–133. 36413381 10.1158/2643-3230.BCD-22-0074 PMC9975770

[219] M. Shi , H. Yuan , Y. Li , Z. Guo , and J. Wei , “Targeting Macrophage Phenotype for Treating Heart Failure: A New Approach,” Drug Design, Development and Therapy 18 (2024): 4927–4942. 39525046 10.2147/DDDT.S486816 PMC11549885

[220] G. Liu , Y. Dai , C. Fu , X. Lv , J. Qin , and J. Xie , “Macrophage Polarization in Myocardial Ischemia‒Reperfusion Injury: Pathophysiology and Therapeutic Targets,” Drug Design, Development and Therapy 19 (2025): 6519–6541. 40766819 10.2147/DDDT.S516001 PMC12323797

[221] C. Chen , R. Wang , X. Chen , Y. Hou , and J. Jiang , “Targeting CD47 as a Novel Immunotherapy for Breast Cancer,” Frontiers in Oncology 12 (2022): 924740. 35860564 10.3389/fonc.2022.924740 PMC9289165

[222] G. Huang and D. Li , “The Macrophage Opera: From Metabolic Prolog to Oncogenic Crescendo in Metabolic Dysfunction‐Associated Steatotic Liver Disease,” Frontiers in Medicine 13 (2026): 1751290. 41788707 10.3389/fmed.2026.1751290 PMC12957107

[223] F. Ingelfinger , S. Krishnarajah , M. Kramer , et al., “Single‐Cell Profiling of Myasthenia Gravis Identifies a Pathogenic T Cell Signature,” Acta Neuropathologica 141, no. 6 (2021): 901–915. 33774709 10.1007/s00401-021-02299-y PMC8113175

[224] A. Ellert‐Miklaszewska , P. Pilanc , K. Poleszak , et al., “7aaRGD—a Novel SPP1/Integrin Signaling‐Blocking Peptide Reverses Immunosuppression and Improves anti‐PD‐1 Immunotherapy Outcomes in Experimental Gliomas,” Journal of Experimental & Clinical Cancer Research 44, no. 1 (2025): 132. 40281508 10.1186/s13046-025-03393-9 PMC12032770

[225] J. Li , X. Jiang , H. Li , M. Gelinsky , and Z. Gu , “Tailoring Materials for Modulation of Macrophage Fate,” Advanced Materials 33, no. 12 (2021): e2004172. 33565154 10.1002/adma.202004172 PMC9245340

[226] M. Dahri , M. Rezaeian , H. Sadeghzadeh , et al., “Nanomaterial‐Driven Macrophage Polarization: Emerging Strategies for Immunomodulation and Regenerative Medicine,” Biomedicine and Pharmacotherapy 190 (2025): 118360. 40712513 10.1016/j.biopha.2025.118360

[227] X. Miao , X. Leng , and Q. Zhang , “The Current state of Nanoparticle‐induced Macrophage Polarization and Reprogramming Research,” International Journal of Molecular Sciences 18, no. 2 (2017): 336. 28178185 10.3390/ijms18020336 PMC5343871

[228] M. Santoni , F. Massari , R. Montironi , and N. Battelli , “Manipulating Macrophage Polarization in Cancer Patients: From Nanoparticles to Human Chimeric Antigen Receptor Macrophages,” Biochimica Et Biophysica Acta‐Reviews on Cancer 1876, no. 1 (2021): 188547. 33932561 10.1016/j.bbcan.2021.188547

[229] R. Cui , J. Zhou , W. Yang , et al., “Ultrasound‐Triggered Nanogel Boosts Chemotherapy and Immunomodulation in Colorectal Cancer,” ACS Applied Materials & Interfaces 17, no. 1 (2025): 211–221. 39660733 10.1021/acsami.4c13358 PMC11783521

[230] Z. Fu , X. Zhao , Q. Zhang , et al., “Synthetic SIGLEC9‐based Chimeric Switch Receptor Augments the Efficacy of CAR Macrophages Against Glioblastoma,” Proceedings of the National Academy of Sciences 123, no. 12 (2026): e2519819123. 10.1073/pnas.2519819123 PMC13012106 41843671

[231] J. Jing , Y. Chen , and E. Chi , “New Power in Cancer Immunotherapy: The Rise of Chimeric Antigen Receptor Macrophage (CAR‐M),” Journal of Translational Medicine 23, No. 1 (2025): 1182. 41152907 10.1186/s12967-025-07115-9 PMC12570453

[232] J. Wei , Y. Dai , N. Zhang , et al., “Natural Plant‐derived Polysaccharides Targeting Macrophage Polarization: A Promising Strategy for Cancer Immunotherapy,” Frontiers in immunology 15 (2024): 1408377. 39351237 10.3389/fimmu.2024.1408377 PMC11439661

[233] K. R. Peterson , M. A. Cottam , A. J. Kennedy , and A. H. Hasty , “Macrophage‐Targeted Therapeutics for Metabolic Disease,” Trends in Pharmacological Sciences 39, no. 6 (2018): 536–546. 29628274 10.1016/j.tips.2018.03.001 PMC5962426

[234] D. H. Kang , Y. Kim , J. H. Lee , H. S. Kang , and C. Chung , “Spatial Transcriptomics in Lung Cancer and Pulmonary Diseases: A Comprehensive Review,” Cancers (Basel) 17, no. 12 (2025): 1912. 40563563 10.3390/cancers17121912 PMC12191356

